# Gas plasma therapy of murine and human diabetic wounds is associated with junctional and hippo signaling and oxidative protein modifications

**DOI:** 10.1016/j.redox.2025.103951

**Published:** 2025-11-28

**Authors:** Anke Schmidt, Kristian Wende, Liane Kantz, Thomas von Woedtke, Sander Bekeschus

**Affiliations:** a ZIK plasmatis, Leibniz Institute for Plasma Science and Technology (INP), Felix-Hausdorff-Str. 2, Greifswald, 17489, Germany; b Institute of Hygiene and Environmental Medicine, Greifswald University Medical Center, Sauerbruchstr., Greifswald, 17475, Germany; c Department of Dermatology, Venerology, and Allergology, Rostock University Medical Center, Strempelstr. 13, Rostock, 18057, Germany

**Keywords:** CAP, Diabetes mellitus, Hippo signaling, Hyperspectral imaging, Human wound exudates, Mass spectrometry, Plasma medicine, Reactive species, ROS/RNS

## Abstract

Impaired diabetic wound healing is characterized by delayed tissue repair due to compromised immune function, reduced angiogenesis and blood flow, and decreased levels of essential growth factors. Gas plasma treatment is an emerging therapeutic approach in redox biology, characterized by its ability to modulate biological processes at different stages of tissue repair. This study examined the wound healing process in a preclinical mouse model of type 2 diabetes (T2DM). Repeated exposure to medical gas plasma has been demonstrated to improve microcirculatory parameters, including superficial tissue oxygenation. Consequently, an optimal wound environment is created. Regulating the interplay between proliferation, the balance of apoptotic pathways, and reactive species generated by gas plasma is critical for several cellular processes. This study revealed that the Hippo signaling pathway and yes-associated protein (YAP) activity play crucial roles in this process. Specifically, these mechanisms were observed to facilitate stage-specific cellular responses in diabetic wounds, significantly affecting the survival of skin cell types. Gas plasma stimulates various biological processes, including cell migration, granulation tissue formation, and collagen synthesis. This stimulation occurs through the remodeling of focal adhesions, the restoration of proper extracellular matrix (ECM) architecture, and the regulation of intercellular junctional structures (e.g., tight, adherens, and gap junctions). Mass spectrometry analysis of gas plasma-treated human wounds revealed that 6 % of the identified proteins had undergone oxidative post-translational modifications, suggesting a link between gas plasma-accelerated healing responses and these modifications. These diverse effects highlight the potential of medical gas plasma as a versatile tool for improving wound management and tissue regeneration in diabetes.

## Graphical abstract

Gas plasma induces changes in signaling pathways relevant to wound healing in a type II diabetic mouse model and diabetic patients. Depending on the stage of wound healing, repeated gas plasma treatment altered the proliferation (e.g., YAP/TAZ signaling) rather than apoptotic cell death on days 9 and 20. It also modulated microcirculatory parameters (e.g., oxygenation, perfusion, and tissue hemoglobin and water indices) and stimulated cell migration and wound granulation by remodeling focal adhesions, the extracellular matrix (ECM), and junctional structures (e.g., tight junctions, adherens junctions, and gap junctions). Oxidative post-translational modifications (oxPTMs) in diabetic foot syndrome (DFS) were investigated using mass spectrometry. BAX, Bcl-2-associated X protein; CDNK1A; cyclin-dependent kinase inhibitor 1A; CTGF, connective tissue growth factor; Cx43, connexin 43; CYR61, cysteine-rich angiogenic inducer 61; FAK, focal adhesion kinase; JAM; junctional adhesion molecule; p53, tumor suppressor protein 53; ROS/RNS, reactive oxygen and nitrogen species; SIRT1, silent mating type information regulation 2 homolog 1; Src, proto-oncogene tyrosine-protein kinase; TAZ, transcriptional coactivator with PDZ binding motif; TEAD, transcriptional enhancer factors; YAP, yes-associated protein; ZO-1/2/3, zona occludens proteins 1/2/3.

**Figure:**
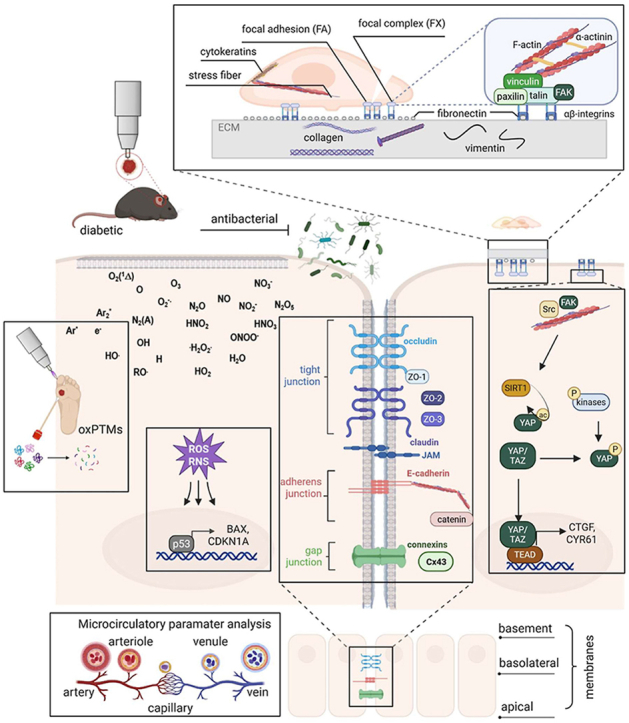

## 1 Introduction

Type 2 diabetes mellitus (T2DM) is a chronic metabolic disorder characterized by the body's inability to effectively utilize insulin, resulting in elevated blood glucose levels. This phenomenon affects millions of individuals worldwide [1]. The increasing prevalence of T2DM poses a considerable challenge to global healthcare systems, with substantial financial resources allocated to the management of diabetes-related complications [2]. Impaired wound healing constitutes a critical concern, as it frequently results in chronic wounds, including diabetic foot ulcers. These ulcers are associated with elevated morbidity and mortality rates, as well as a significant risk of infection and amputation [3]. The pathological mechanisms underlying delayed wound healing in T2DM involve complex interactions of hyperglycemia-induced oxidative stress, chronic inflammation, vascular dysfunction, increased susceptibility to infection, and impaired cellular responses such as fibroblast migration and proliferation [4,5]. Reduced levels of healing factors or impaired activity of signaling pathways have been shown to contribute to this process [6].

A plethora of treatment options exists for diabetic wounds, including wound debridement and dressings, growth factor treatment, hyperbaric oxygen therapy, negative pressure wound therapy, electrical stimulation, nutritional support, medications, and surgery [7,8]. The aforementioned treatment options have been meticulously designed to promote wound healing by creating a moist environment that is conducive to angiogenesis and the formation of granulation tissue. The objective of this approach is to mitigate inflammation and expedite the removal of excess wound fluid. These strategies frequently yield suboptimal outcomes, underscoring the necessity for innovative technologies. A promising research avenue involves the utilization of medical gas plasma, which is defined as an ionized gas comprising reactive oxygen and nitrogen species (collectively termed as ROS/RNS) [9]. Gas plasma has been demonstrated to possess the capacity to modulate model proteins through oxidative post-translational modifications (oxPTM) [10,11] and cellular processes that are imperative for wound healing by establishing an environment conducive to angiogenesis [12], as shown *in vitro* [13,14], and in rodents [[15], [16], [17]]. The observed effects are, at least in part, attributable to gas plasma-generated ROS/RNS and redox signaling responses [18], which have been demonstrated to affect the biological activity of model protein signaling pathways and improve microcirculation in blood vessels [19]. Hyperspectral imaging (HSI) is a non-invasive imaging technology that captures a spectrum of light at multiple wavelengths to provide detailed information about the biochemical and physiological status of tissues [[20], [21], [22]]. HSI provides data on oxygenation, perfusion, tissue composition, and inflammation in the wound bed. This information can aid in assessing wound healing, determining tissue viability, and guiding treatment. Furthermore, mass spectrometry was employed to investigate oxPTMs. This method facilitates the precise identification and characterization of functional modifications, thereby contributing to a more profound comprehension of their biological roles and implications.

The dysregulation of multiple signaling pathways has the potential to impede the healing process in diabetic wounds. In addition to its direct antimicrobial properties [[23], [24], [25]], gas plasma therapy activates the transcription factor Yes-associated protein (YAP) [[26], [27], [28]] and stimulates the formation of granulation tissue [29,30]. It has also been demonstrated to facilitate the remodeling of intercellular (tight) junctions [31] and improve the structural integrity of healing tissue. In wounds afflicted by type 1 diabetes, the Hippo signaling pathway is often dysregulated. These phenomena have the potential to hinder the healing process. This multifaceted mechanism of action positions gas plasma as a versatile and effective tool for addressing the challenges of diabetic wound healing. Despite continuous improvements and refinements in cell culture-based applications, *in vivo* mouse models have been utilized to investigate the mechanisms and healing pathways of diabetic wound healing [32,33]. Establishing causality in humans poses a significant challenge. To address this challenge, a T2DM mouse model was utilized, characterized by leptin deficiency, which leads to persistent obesity [34], accompanied by multiple metabolic alterations, including hyperlipidemia, temperature regulation defects, and decreased physical activity [35].

Researchers are exploring various strategies to improve diabetic wound healing, which is a significant concern due to reduced blood flow, neuropathy, and impaired immune function. In this study, we investigated the potential of gas plasma therapy to assess four distinct microcirculatory parameters and to modulate the Hippo pathway and YAP activity in a mouse model of T2DM. We demonstrate that multiple targets are concurrently regulated in gas plasma-assisted wound healing, encompassing the expression of structural proteins, ECM components, and junctional proteins. Human samples isolated from gas plasma-treated diabetic wounds were tested for key targets and oxidative post-translational modifications, confirming the aforementioned findings. The present study explores the potential of gas plasma as a novel therapeutic approach for treating diabetic wounds.

## 2 Materials and methods

### 2.1 Animals and wounding

To facilitate the analysis of wound healing, eight-week-old mice of the B6.Cg-Lepob/J strain (commonly referred to as Lepob or diabetic; Charles River Laboratories, Germany) were employed in the experiments, which were performed according to the study design approved by the responsible ethical committee (Mecklenburg-Vorpommern, Germany; approval code: 7221.3-1-044/16). The protocols were also in accordance with the guidelines delineated in the NIH Guide for the Care and Use of Laboratory Animals. The mice were housed under a 12-h light/dark cycle, with access to food and water provided ad libitum. The B6.Cg-Lepob/J model is characterized by hyperglycemia, with blood glucose levels fluctuating between 250 and 600 mg/dL, and triglyceride levels exceeding 150 mg/dL, according to the vendor (Charles River Laboratories). The excision of the upper epidermal and dermal layers was performed on both ears using microscissors, as previously described in studies [36]. The full-thickness dermal wounds measured approximately 4 mm squared. For subsequent analyses, tissue samples were obtained from the wounds on days 9 and 20 after the injury.

### 2.2 Gas plasma treatment of diabetic mouse wounds and skin cells

The kINPen MED atmospheric pressure argon plasma jet (neoplas MED, Germany) was used for wound treatment. The device operated at a frequency of 1 MHz and an electric potential of 2–6 kV. Argon gas (purity 99.9999 %, supplied by Air Liquide, Germany) was utilized at a flow rate of 5 standard liters per minute (slm). The plasma jet, which has been approved as a Class IIa (today, class IIb) medical device in Germany and throughout Europe since 2013 37], was applied in the conductive mode [38], in which the gas plasma effluent directly contacted the wounded tissue. The standardization of treatment was achieved by maintaining an 8 mm distance using a sterilizable spacer. The treatment of ear wounds involved two distinct approaches: the first approach entailed exposing the wounds to gas plasma for 10 s every third day, while the second approach entailed omitting any treatment (controls, ctrl). The mice were divided into eight groups based on their sex, with each group containing four to five animals. The study spanned either nine days (comprising four gas plasma treatment sessions) or 20 days (comprising six gas plasma treatment sessions) following wounding, and the results were then compared with those of the corresponding untreated controls. A recently published study examined treating ear wounds with argon gas alone, rather than with the excited species generated by gas plasma [39]. Several studies have confirmed the lack of biological effects of pure argon gas using the kINPen jet [11,40,41]. Skin cells (e.g., dermal fibroblasts and keratinocytes) were plasma-treated for 20 s, 60 s, and 180 s and compared to untreated controls (ctrl) as previously described [17].

### 2.3 Hyperspectral imaging of diabetic wounds

The hyperspectral imaging camera system *TIVITA Tissue* (Diaspective Vision, Germany) was used for real-time visualization of wounds and quantification of microcirculatory parameters [42]. The visible and the invisible spectra (500–1000 nm) were both analyzed, and the resulting spectra were mapped to different characteristics, such as oxygenation (StO_2_) in superficial skin layers and the oxygen saturation and perfusion in deeper layers (4–6 mm) using the near-infrared (NIR) index. The distribution of hemoglobin within the microcirculatory system, as well as the water content, were determined using the tissue hemoglobin index (THI) and tissue water index (TWI). Hyperspectral images were obtained under controlled conditions, with a 50 cm distance maintained between the subject and the camera. All parameters were recorded immediately after wounding and gas plasma treatment and compared to those of untreated controls (set to 1) every third day until the endpoint (d20). For the distinct parameters, the wound area was marked with circles (red), as indicated, and all parameters were calculated independently using the TIVITA Suite Wound software (n ≥ 4). The resulting documentation yielded three measurements for each of the four parameters and 16 wounds on four days (d0, d3, d6, d9) and 18 wounds on three additional days (d12, d16, d20). The total number of measurements collected was 1488.

### 2.4 Gas plasma treatment of human diabetic wounds

The study's sample population included 18 patients diagnosed with diabetic foot ulcers and/or diabetic foot syndrome (DFS, Table A1). This selection was made after provision of informed consent and in accordance with the ethical approval granted by the local ethics committee (BB047/22). The management of diabetic wounds involves the application of a disinfectant solution, such as octenidine dihydrochloride, phenooxyerhanol, polyhexanide, or hypochlorous acid, for the purpose of rinsing the affected area. A sterile curette was utilized to excise fibrin deposits, small necrotic regions, and macerated tissue, thereby refreshing the wound edges. Subsequent to the completion of the final disinfection of the wound, wound exudates were collected prior to and following standard wound care, with and without the incorporation of gas plasma (ctrl = before plasma treatment, gas plasma group = after plasma treatment). The collection of exudates was performed using sterile FLOQSwabs (Copan, Italy), with the gas plasma effluent being applied to the wound in a meandering pattern (*Essener Kreisel*) at a rate of approximately 10 s per square centimeter.

### 2.5 Gene and protein expression of homogenized mouse and human tissue samples

Mouse-ear tissues were harvested from the right ears on days 9 and 20 after injury. For subsequent analyses, the tissues were immediately snap-frozen in liquid nitrogen and stored at −80 °C. Subsequently, the samples were then subjected to homogenization using either RNA lysis buffer (Bio&Sell, Germany) for gene expression studies or RIPA buffer supplemented with protease and phosphatase inhibitors (cOmplete Mini, phosSTOP, PMSF; Sigma-Aldrich, Germany) for protein validation experiments. For the quantitative polymerase chain reaction (qPCR) analysis, primary diabetic wound exudates were collected from diabetic patients receiving either single (n = 5) or multiple gas plasma treatments (n = 4). Additionally, untreated wound exudate (n = 9) was collected from each patient prior to plasma treatment. Swabs were placed in tubes containing 500 μL of RNA lysis buffer (Bio&Sell, Germany), which was subsequently kept on ice. Following the homogenization of the murine and human samples using a FastPrep-24 5G homogenizer (MP Biomedicals, Germany), and after lysis of skin cells in RNA lysis buffer, total RNA was isolated using RNA kit (Bio&Sell, Germany). qPCR was performed to determine mRNA expression levels. A total of 1 μg of RNA was converted to cDNA, and qPCR was conducted in duplicate using SYBR Green I Master (Roche Diagnostics, Switzerland) together with gene-specific primers (BioTez, Germany) for mouse (Table A2) and human genes (Table A3). *GAPDH* and *RPL13A*, which demonstrated no alterations in response to gas plasma treatment, were selected as internal reference genes for normalization purposes. The 2^−ΔΔCT^ method was employed to assess the levels of gene expression. To corroborate the qPCR findings at the protein level, key markers of cellular response were analyzed using mouse tissue. The present analysis included components of the Hippo signaling pathway, such as Yes-associated protein (YAP), transcriptional coactivator with PDZ-binding motif (TAZ), and phosphorylated YAP (phospho-Ser127). Furthermore, the present study evaluated proteins associated with cellular adhesion and structural integrity, including focal adhesion kinase (FAK), vinculin (VCL), vimentin (VIM), E-cadherin, and β-catenin. GAPDH was utilized as the reference protein (all antibodies from Cell Signaling Technology, Germany). The quantification of protein expression levels was performed using the WES system (ProteinSimple, Germany) with Compass software (ProteinSimple, Germany), following the manufacturer's protocol. Protein levels were expressed as fold changes (FC) relative to the respective controls.

### 2.6 Histology, immunohistochemistry, and immunofluorescence analysis

The wounded areas of the left ears were meticulously excised and fixed in 4 % paraformaldehyde (Sigma-Aldrich, Germany) for 24 h on days 9 and 20 following the initial injury. Subsequently, the samples were then embedded in paraffin and sectioned at 5 μm using a microtome. The tissue sections were subjected to hematoxylin and eosin (H&E; Carl-Roth, Germany) staining, and collagen fibers were subsequently visualized through picrosirius red staining (PSR, Direktrot 80; Sigma-Aldrich, Germany). These procedures were carried out in accordance with well-established protocols [43]. For immunohistochemical analysis, tissue sections were incubated with primary antibodies directed against specific proteins. The detection of these sections was subsequently facilitated by the utilization of SignalStain Boost IHC reagents (Cell Signaling Technology, Germany). Subsequently, the tissue sections underwent a washing step, followed by permeabilization with 0.01 % Triton X-100 in phosphate-buffered saline (PBS; Sigma-Aldrich, Germany). Subsequently, the sections were incubated with primary antibodies targeting various markers, including PCNA (proliferation), TUNEL and p53 (apoptosis), p53 (transcription factor), and iNOS (all from Cell Signaling Technology, Germany). Secondary antibodies conjugated to Alexa Fluor 488 or 594 (Life Technologies, Germany) were used for fluorescence detection, and DAPI was used to counterstain cell nuclei. The stained tissue sections were mounted on glass slides using VectaShield mounting medium (Biozol, Germany) and then examined using an Axio Observer Z.1 fluorescence microscope (Zeiss, Germany).

### 2.7 Protein oxidation mapping in human chronic diabetic wounds

Following the sampling of DFS wound exudates, swabs were placed in 1 mL ice-cold phosphate-buffered saline lacking calcium and magnesium, 5 mM EDTA, and miniComplete protease/phosphatase inhibitor (Roche, Germany). In instances where such samples were deemed necessary, they were stored at −80 °C. For the workup, samples were thawed on wet ice, vortexed, and treated for 10 s with a tip sonicator (Sartorius Labsonic M, Germany). After centrifugation (15 min, 21,000×*g*, 4 °C), the proteins present in the transparent upper layer of the solution were precipitated by the addition of acetone at −20 °C and incubated overnight. The precipitate was subsequently pelleted and washed with a solution of cold 80 % acetone in water. Following this step, the protein pellet was allowed to dry for 5 min. Subsequently, 100 μg of protein was processed and digested using S-trap mini columns (ProtiFi, USA) according to the recommended procedure. Briefly, proteins were solubilized in 5 % sodium dodecyl sulfate, reduced (20 mM dithiothreitol final), alkylated (40 mM iodoacetamide final), and trapped on the S-trap columns. Following the completion of the washing step, the digestion process was initiated using 10 μg of sequencing-grade trypsin (Promega, Germany) at 37 °C for 18 h. The eluted peptides were then desiccated in glass microvials (VWR, Germany) using a concentrator (Eppendorf, Germany) and reconstituted in 20 μL of buffer A (0.1 % acetic acid in water). Subsequently, the samples were subjected to reversed-phase column chromatography (PepMap 75 μm × 25 cm C18, with PepMap 100 μm × 2 cm C18 desalting trap column; ThermoFisher, Germany) on a Dionex UltiMate 3000 RSLCnano HPLC system (ThermoFisher, Germany) connected to an Exploris 480 mass spectrometer (ThermoFisher, Germany). The separation was achieved by employing a 65-min gradient of 0.1 % acetic acid in water (A) and 95:5 acetonitrile:0.1 % acetic acid (v/v; B) with a gradient of 4–45 % buffer B at a flow rate of 300 nL/min. The Exploris 480 mass spectrometer (MS; ThermoFisher, Germany) was operated in data-dependent acquisition (DDA) mode, with positive polarity, at a capillary temperature of 250 °C and a spray voltage of 2 kV. Peptides were analyzed with one full scan (350–1200 m/z, R = 120,000 at 200 m/z) with a target of 5 × 10^3^ ions, followed by 15 data-dependent MS2 scans with higher energy collision dissociation (HCD). The maximum injection time (IT) was set to 50 ms (ms), the isolation width was set to 1.0 m/z, the normalized collision energy (NCE) was set to 30 %, and the detection was set in Orbitrap mode (R = 15,000 at 200 m/z). The dynamic exclusion function was enabled and set to 30 s. The raw data were then subjected to analysis using Proteome Discoverer (version 2.4.1.55; Thermo Scientific, USA), which was run against the UniProt FASTA file. UP000005640_9606 (*Homo sapiens*) contains 11,395,384 residues, as determined by MSAmanda, Sequest HT, and PMI-Byonic (v 5.2.5). The search engine parameters for MSAmada (and Sequest HT) were set as follows: peptide mass tolerance = 10 ppm; fragment mass tolerance = 0.03 (0.02) Da; cleavage specificity = trypsin; missed cleavage sites = 2; (static modifications = cysteine carbamidomethylation) and variable modifications = tyrosine phosphorylation, cysteine trioxidation, and N-Terminus acetylation. The MS2 search engine results underwent a filtration process that targeted a 1 % false discovery rate (FDR) on protein and peptide levels, employing the Percolator node as the filtration mechanism. The utilization of MSAmanda and Sequest HT nodes facilitated the identification of proteins. At the same time, label-free quantification (LFQ) data were obtained through the implementation of the Minora feature Detector node. PMI-Byonic was used to identify oxidative modifications (oxMod) based on a modification list derived from prior knowledge of gas plasma-induced protein oxidation [44,45]. The following settings were implemented: peptide and fragment mass tolerance = 10 ppm; and cleavage specificity = trypsin with a maximum of two missed cleavages and one modification per peptide. To facilitate further analysis, lists with peptide spectral matches (PSMs) created by the Byonic node were filtered for a Byonic Score of at least 500 and high peptide confidence. These lists were then imported into MS Excel and Perseus (version 2.0.6.0) [46] for additional data analysis. The presence of oxidative modifications was deemed valid if such modifications were observed in at least 70 % of the treatment group samples. An oxMod was designated as novel if it was not present in either treatment group at a frequency of ≥70 % while concurrently not present in ≥70 % of the control group.

### 2.8 Statistical analysis

All experiments were conducted on tissues from a minimum of five wounds per group. The *in vitro* assays were repeated independently in triplicate. The results are expressed as the mean ± standard deviation (S.D.), unless otherwise indicated. Statistical comparisons were conducted with significance levels denoted as ∗p < 0.05, ∗∗p < 0.01, and ∗∗∗p < 0.001. The generation of graphs and statistical analyses was performed using Prism 9.51 (GraphPad Software, USA). Unpaired Student's t-test was used for comparisons between two groups, while the one-way ANOVA was employed for analyses involving multiple groups. For qPCR data, significance values are displayed in the graphs only if they are less than 0.67 or greater than 1.5 (indicated by dashed lines).

## 3 Results

### 3.1 Gas plasma-treated wounds altered angiogenic factors

**Fig. 1:**
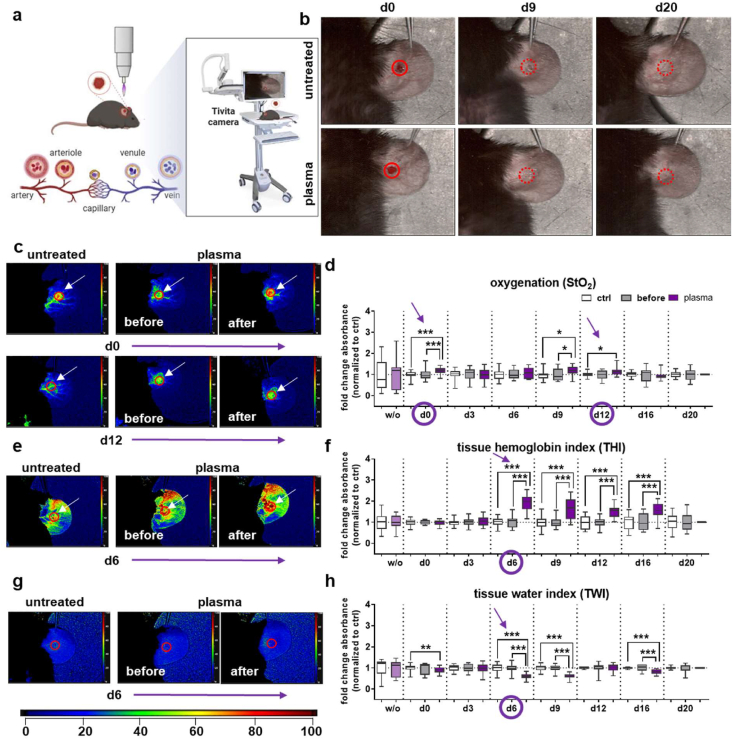
**Non-invasive hyperspectral imaging (HSI) of diabetic wounds in female mice. (a)** Study overview of HSI after gas plasma treatment. **(b)** Representative RGB images captured by an HSI camera (Tivita) highlight the wounds in the untreated control (top image) and the gas plasma-treated ear (bottom image) at d0, d9, and d20. (**c**) Hyperspectral images of StO_2_ at d0 (top panel) and d12 (bottom panel), showing untreated controls on the left and a gas plasma-treated wound (middle and right images). (**d**) Measured StO_2_ data over time (d0-d20). (**e**) Hyperspectral images of THI for the untreated control (left image) and a gas plasma-treated wound (middle and right images) at d6. (**f**) Measured THI data over time (d0-d20). (**g**) Hyperspectral images of NIR for the untreated control (left image) and a gas plasma-treated wound (middle and right images) at d9. (**h**) Quantification of NIR values over time (d0-d20). (**i**) Hyperspectral images of TWI for the untreated control (left image) and a gas plasma-treated wound (middle and right images) at d6. (**j**) Quantification of TWI water content over time (d0-d20). Points of interest were placed on the wound area (red circle and arrows in c, e, g, i). The color scale ranges from blue (0) to red (100 %). Days of interest were shown as indicated (purple arrow in d, f, h, j). StO_2_, tissue oxygenation; THI, tissue hemoglobin index; NIR, near-infrared index; TWI, tissue water index. The total number of measurements collected was 748 (n ≥ 4). d, days; w/o, without wounding.

As previously described, a comparative analysis was conducted on the healing responses, as measured by wound healing indexes, among the sexes within the gas plasma-treated and the untreated control groups [47]. Gas plasma treatment has been shown to enhance re-epithelialization and reduce wound closure time in both female (Fig. A1a) and male diabetic (Fig. A1b) and non-diabetic wild-type C57BL/6J mice at each evaluation time point [47]. To obtain detailed information about the biochemical and physiological status of the wounded tissue, microcirculatory wound parameters were monitored. The parameters encompassed superficial tissue oxygenation (StO_2_), perfusion, and deep tissue oxygenation (NIR, near-infrared index), hemoglobin distribution (THI), and water content (TWI), which were induced by gas plasma in diabetic mice (Fig. 1a). Initially, an ear wound (d0) was created. No size disparities were detected between the untreated and gas plasma-treated wounds (see left images in Fig. 1b). Our “before plasma” values resemble the baseline reference point prior to each treatment. This indicates that the system recovers between sessions, which supports the idea that multiple treatments are necessary for long-lasting results. Subsequently, a comparative analysis was conducted between plasma-supported wound healing (bottom panel) and untreated wounds (ctrl, top panel) over nine or twenty days (middle and right images in Fig. 1b). This analysis revealed the healing status of both groups of females. The study found that nearly complete wound closure and maturation of the epidermal and dermal layers were achieved at d20 (red circles, Fig. 1b). Subsequent to gas plasma treatment, StO_2_ tended to increase, manifesting at levels that were considerably higher in gas plasma-treated wound areas at d0, d9, and d12 (purple arrows as corroborated by the representative images at d0 and d12 in females (Fig. 1c)) (Fig. 1d). A significant increase in hemoglobin distribution (THI) was observed from d6 to d16, as evidenced by representative hyperspectral images of the untreated wound (ctrl) compared to the gas plasma-treated wound (before and after gas plasma treatment, Fig. 1e–f). Oxygen saturation in deeper layers was consistently lower compared to the untreated control (ctrl) on all days measured, as shown in a representative image at d9 (Figs. A1c–d), suggesting gas plasma-induced changes in the scattering properties of the skin in collagen composition, a chromophore that absorbs NIR light, and/or melanin content, leading to a decrease in NIR reflectance. Furthermore, the skin contains water, which exhibits pronounced absorption peaks in the NIR range (>900 nm). TWI demonstrated a persistent decline in gas plasma-treated wounds from days 0–16 (Fig. 1g–h). This finding suggests potential alterations in membrane permeability, tissue structure (e.g., collagen and extracellular matrix composition), and/or the absorption spectrum. These alterations may be attributable to the augmented presence of oxidative by-products.

**Fig. 2:**
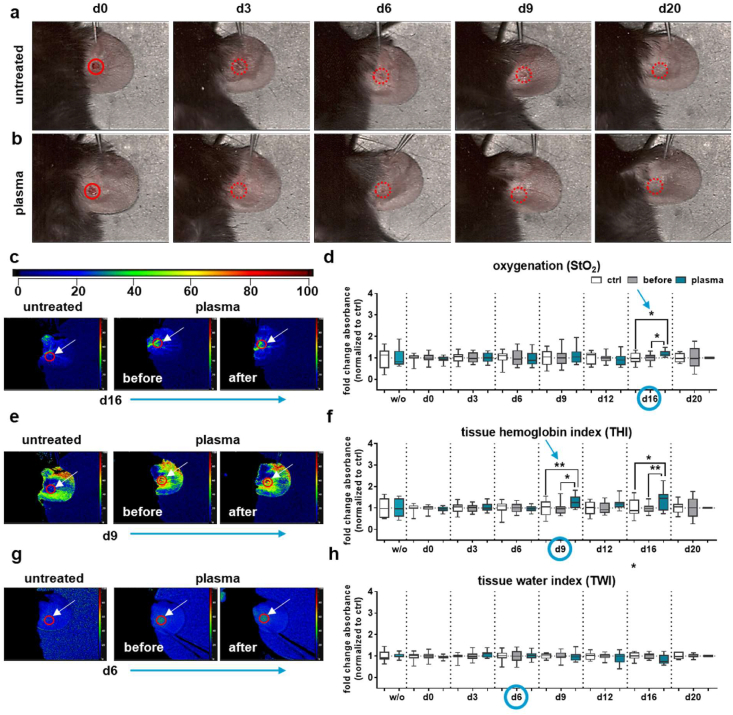
**Non-invasive hyperspectral imaging of diabetic wounds in male mice. (a**–**b)** Representative RGB images acquired with the HSI camera highlighting wound healing in untreated controls (**a**) and gas plasma-treated ears (**b**) at days 0, 3, 6, 9, and 20 (red circle). (**c**) Hyperspectral images of StO_2_ for the untreated control (left image) and a gas plasma-treated wound (middle and right images) at d16. (**d**) Measured StO_2_ data over time (d0-d20). (**e**) Hyperspectral images of THI for the untreated control (left image) and a gas plasma-treated wound (middle and right images) at d9. (**f**) Measured data of THI over time (d0-d20). (**g**) Hyperspectral images of NIR for the untreated control (left) and a gas plasma-treated wound (middle and right images) at d0 (top panel) and d9 (bottom panel). (**h**) Quantification of NIR values over time (d0-d20). (**i**) Hyperspectral images of TWI for the untreated control (left image) and a gas plasma-treated wound (middle and right images) at d6. (**j**) Quantification of TWI water content over time (d0-d20). Points of interest were placed on the wound area (red circle and arrows in c, e, g, i). The color scale ranges from blue (0) to red (100 %). Days of interest were shown as indicated (cyan arrow in d, f, h, j). StO_2_, tissue oxygenation; THI, tissue hemoglobin index; NIR, near-infrared index; TWI, tissue water index. The total number of measurements collected was 740 (n ≥ 4). d, days; w/o, without wounding.

Subsequently, the gas plasma-assisted wound closure process in males was compared to untreated wounds over 20 days (red circles, Fig. 2a), revealing enhanced healing outcomes following gas plasma exposure (red circles, Fig. 2b). Nonetheless, we have documented disparities in microcirculatory parameters between the sexes. The levels of oxygen saturation in the superficial layers attained their zenith at d16 in gas plasma-treated wounds (cyan arrow, Fig. 2c–d), while the distribution of hemoglobin (THI) persisted at elevated levels from d9 to d16 (Fig. 2e–f). NIR values were consistently lower on days 0 and 9 (Fig. A1f), as shown in a representative image at d9 (Fig. A1e). No differences in water content after gas plasma treatment were observed for the TWI parameter (Fig. 2g–h). Nitric oxide (NO) produced by iNOS plays a crucial role in regulating microcirculatory blood flow by relaxing the smooth muscle in the vessel walls. Hyperspectral imaging captures these changes as dynamic variations in blood volume and velocity. We also validated that gas plasma induced significantly higher expression of the inducible isoform of nitric oxide synthase (iNOS, gene name *NOS2*) at d9 in diabetic wounds by qPCR (Figs. A2a–b).

### 3.2 Gas plasma-treated wounds alter skin cell proliferation, apoptotic events, and Hippo signaling

**Fig. 3:**
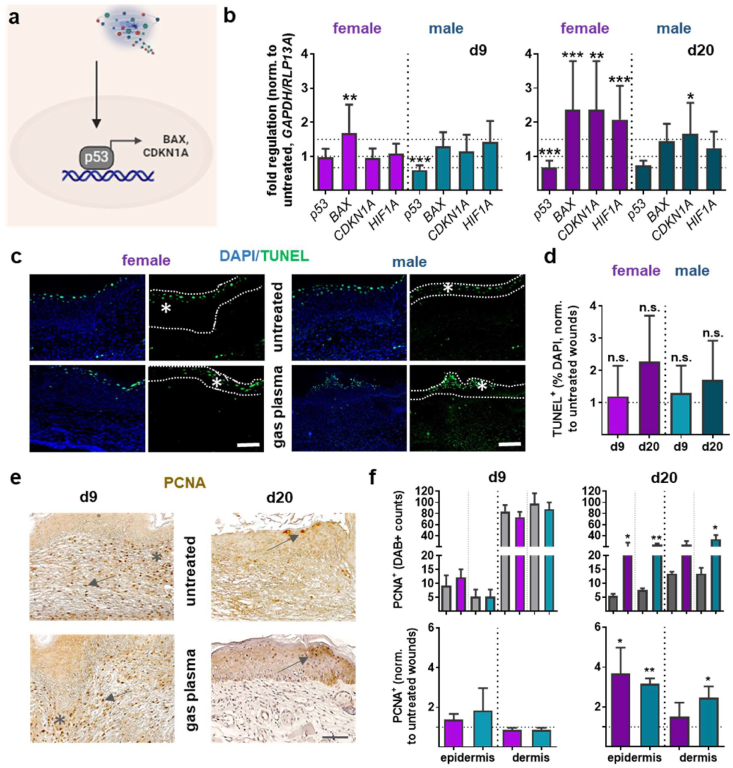
**Changes in skin cell proliferation and apoptotic events after gas plasma treatment in diabetic wounds. (a)** The scheme illustrates the signaling process involving p53**. (b)** qPCR-based expression of p53 signaling targets, including *p53* itself, *BAX* (BCL-2-associated X protein), cyclin-dependent kinase inhibitor 1A (*CDKN1A* or p21), and the hypoxia-inducible factor (*HIF*) 1A, was conducted using total RNA isolated from the wound regions at both endpoints (d9/d20). **(c)** TUNEL staining of apoptotic cells (green) in female (left) and male (right) mice quantified within the white-dashed region; nuclei were stained with DAPI (blue). **(d)** The quantification of TUNEL-positive cells (white star in c) was done at both endpoints. **(e)** Immunohistochemical staining for proliferating cell nuclear antigen (arrows, PCNA); postoperative photos from d9 and d20 are shown, comparing gas plasma-treated with untreated wounds. **(f)** The number of PCNA-positive cells in the wounds was quantified at both endpoints (d9 left, d20; right) as absolute PCNA-positive counts (top graphs, grey columns: untreated ctrl) and after normalization to untreated wounds (bottom graphs). Female and male mice were used on d9 and d20 (n > 4)**.**

While basal keratinocytes and fibroblasts accomplish re-epithelialization and dermis replacement through cell migration and proliferation, the assertion that a coordinated process of proliferation and apoptosis is critical during wound healing is a well-established concept in the field of wound healing research. To validate the occurrence of apoptotic events, the transcriptional modulation of p53 signaling was examined as a crucial regulator of cell cycle arrest and apoptosis, also known as programmed cell death, in response to various cellular stresses (Fig. 3a). Using qPCR, the expression pattern of the p53 tumor suppressor gene was found to be significantly downregulated (with exception of females) on d9. Bcl-2-associated X gene (*BAX*), a cofactor of p53, plays an essential role in the regulation of the intrinsic, mitochondrial apoptotic cell pathway. *BAX* levels were higher after gas plasma treatment compared to untreated wounds. The cell cycle-related cyclin-dependent kinase inhibitor 1A (*CDKN1A*) exhibited no alterations on day 9; however, it demonstrated a substantial increase on day 20. The observed increase in hypoxia-inducible factor 1α (*HIF1A*) expression suggests regulation by a redox-sensitive mechanism (Fig. 3b). The ROS/RNS generation by gas plasma has been demonstrated to be a potent instrument for the treatment of diabetic wounds, particularly in the context of the body's response to either modulate biological responses or counteract bacterial infection (Fig. A2c). As demonstrated in this study, gas plasma induced the expression of several defensins (e.g., *DEFB1, DEFB6, DEFB26, DEFB28, DEFB50*) and lysozymes (e.g., *LYZ1/2*) in females (Fig. A2d), indicating a potential gas plasma-mediated stimulation of antimicrobial peptide (AMP) production, which could enhance antimicrobial properties of treatment. By contrast, no significant antibacterial response was observed for *DEFB1/6* and *LYZ1/2* expression in plasma-treated males compared to untreated controls at d9 (only a tendency was observed at d20). These results suggest that the immune response may be modulated by sex-dependent factors, as demonstrated by the gender-specific differences in AMP expression, highlighting the importance of considering gender as a biological variable in studies of innate immunity.

To analyze the nature of cell death, terminal deoxynucleotidyl transferase dUTP nick end labeling (TUNEL) staining of apoptotic cells was employed, as illustrated in the representative images on day 20 in epidermal layers (regions between dashed lines, Fig. 3c). For both endpoints, only slight differences in the number of apoptotic cells were found after quantification, indicating either the apoptosis of immune cells, such as neutrophils and macrophages, which occurs during the resolution of inflammation in the early stages of wound healing (d9), or the apoptosis of specific cells, such as excessive fibroblasts or endothelial cells, to prevent overgrowth in the proliferative phase and tissue remodeling (d20, Fig. 3d). Subsequently, the application of gas plasma treatment resulted in a substantial augmentation in the rate and extent of cell proliferation during the reparative process, as evidenced by the staining of proliferating cell nuclear antigen (PCNA). At d9, PCNA-positive cells were predominantly located in the dermis in both groups, as shown in representative immunohistochemical images (left images, Fig. 3e) and quantitative analysis (left graphs, Fig. 3f). Nevertheless, we observed only minor variations in gas plasma-treated skin tissue compared to the untreated control at this endpoint. Conversely, PCNA-positive cells were detected to a lesser extent in the epidermal and dermal layers of the defect areas at d20 after wounding, as shown in representative staining (right images, Fig. 3e). However, in comparison with untreated wounds, a partial yet significant increase in PCNA-positive cells were observed on d20 in the gas plasma-treated epidermal and dermal layers of wounded diabetic skins (right graphs, Fig. 3f), thereby confirming the active division of skin cells and their contribution to the formation of new layers.

**Fig. 4:**
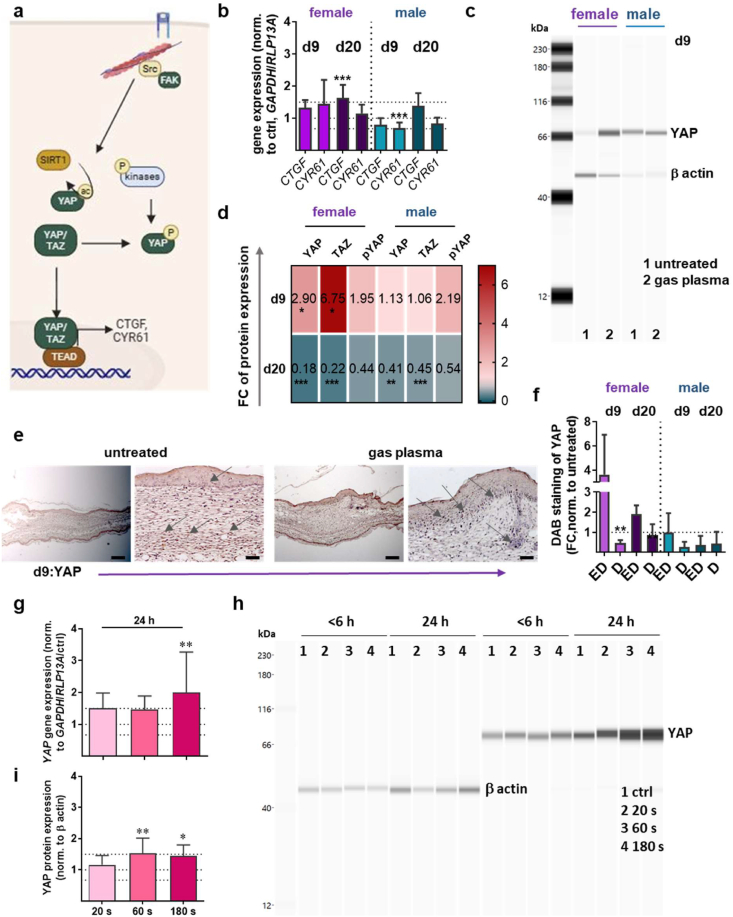
**Hippo signaling after gas plasma treatment in diabetic wounds (a)** The visual representation illustrates the Hippo signaling pathway, which is responsible for regulating the expression of YAP/TAZ. **(b)** qPCR-based expression analysis of connective tissue growth factor (*CTGF*) and cysteine-rich angiogenic inducer 61 (*CYR61*)**. (c)** Representative image of YAP protein expression after gas plasma-treatment in both female and male mice, in comparison to the untreated control group at d9. The β-actin protein was utilized as a housekeeping control protein. **(d)** YAP, TAZ, and pYAP were quantified by WES analysis. (e) Representative images of immunohistochemical staining of YAP at d9 in females in untreated (left) and gas plasma-treated wounds (right photos), and **(f)** their quantification in untreated vs. gas plasma-treated epidermis (ED) and dermis **(D)** at d9 and d20 in both sexes. **(g)** qPCR-based expression analysis of YAP 24 h after plasma treatment of cultured skin cells. **(h)** Representative image of YAP protein expression 6 and 24 h after gas plasma-treatment (20 s, 60 s, 180 s) in comparison to the untreated data obtained in skin cells (ctrl). The β-actin protein was utilized as a housekeeping control protein. (i) YAP was quantified by WES analysis. Data are expressed as mean ± S.D.; ∗p<0.05, ∗∗p<0.01, and ∗∗∗p<0.001 compared with untreated controls; scale bar are 200 μm (left images for untreated and gas plasma) and 50 μm (right images for untreated and gas plasma) in e; female and male mice were used on d9 and d20 (n > 4). FC, fold change**;** SIRT1, silent mating type information regulation 2 homolog 1; Src, proto-oncogene tyrosine-protein kinase; TAZ, transcriptional coactivator with PDZ binding motif; TEAD, transcriptional enhancer factors; YAP, yes-associated protein.

Given the potential of modulation of YAP (Yes-associated protein) and TAZ (transcriptional coactivator with PDZ-binding motif) activity (Fig. 4a) to enhance cell proliferation and tissue regeneration, the present study sought to investigate the cellular processes and components of Hippo signaling through gene and protein expression analysis. The quantification of the connective tissue growth factor (*CTGF*) and cysteine-rich 61 (*CYR61*) by qPCR, which are key downstream targets and are regulated after nuclear binding of YAP and TAZ, exhibited overexpression in females at d9, which was significant for CTGF on d20. This is in contrast to males, where both levels were decreased with exception for CTGF on d20 (Fig. 4b). Other downstream targets of YAP, including transforming growth factor (TGF) β1 [47,48[47,48], vascular endothelial growth factor (VEGF) [47,49], and nuclear factor erythroid 2-related factor 2 (Nrf2) [47,50], were found to be upregulated by gas plasma in our diabetic mouse model. Using WES analysis, we observed a significant upregulation of YAP protein expression in females at d9, as shown in a representative WES image (Fig. 4c). The upregulation of YAP was transient, as evidenced by a decrease in YAP expression at the second endpoint compared to the untreated control (Fig. A3a). Furthermore, the quantification of YAP/TAZ and pYAP (phospho-Ser127) protein expression at both endpoints showed a transient increase that was abolished at d20 in females (Fig. 4d–A3b). On d20, we observed significant downregulation of YAP and TAZ proteins in both sexes; however, this was not significant for pYAP (Fig. 4d). The upregulation of YAP in female mice was confirmed by immunohistochemical staining (brown dots, Fig. 4e) in the epidermis (ED) in contrast to the dermis (D) of wounded tissue at d9 (Fig. 4f). YAP levels in the epidermis of male mice were similar to those of the untreated controls. However, YAP levels decreased in the dermis, as was also shown for d20 in both layers. To demonstrate plasma-induced activation of YAP, we treated various skin cells (e.g., dermal fibroblasts and keratinocytes) *in vitro* with different plasma treatment times. We observed a time-dependent increase in YAP gene expression, which was particularly significant after 180 s (Fig. 4g). This result was validated by analyzing the expression of YAP protein. As shown in Fig. 4h, a representative image of a WES analysis revealed a significant increase in YAP protein 24 h after plasma treatment, which was confirmed by quantifying the protein bands (Fig. 4i). In contrast, no changes were detectable up to 6 h after plasma treatment. Similarly, an increase in TAZ activity was observed (left diagram, Fig. A3c). Although CTGF tended to increase 24 h after plasma treatment, CYR61 was significantly elevated after 180 s of plasma treatment. Nevertheless, YAP/TAZ-independent regulation could also be identified here, as both targets were significantly more strongly expressed after a short plasma treatment time of 20 s than in the untreated control (Fig. A3c).

### 3.3 Gas plasma-treated diabetic wounds showed specific patterns of structural protein expression

**Fig. 5:**
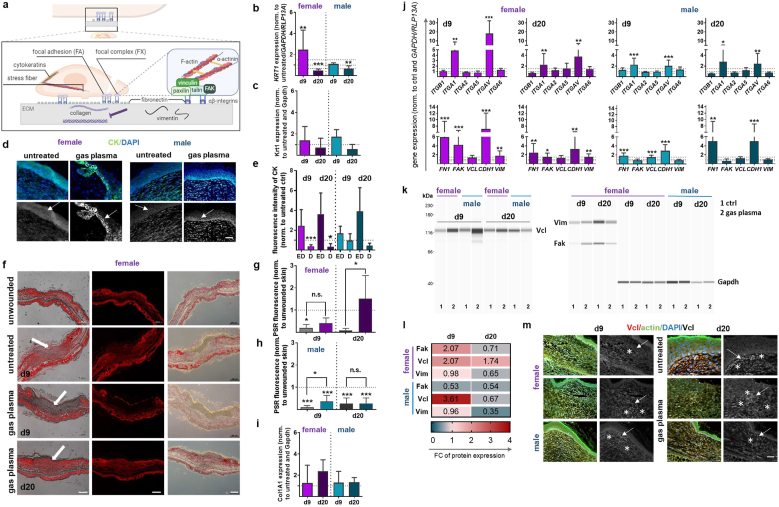
**The interplay between focal adhesions and gas plasma-generated ROS**/RNS **is critical for cellular adhesion and migration that occurs during wound healing.** (**a**) Focal adhesions (FAs) are essential for cell migration; they dynamically form and disassemble as keratinocytes and fibroblasts migrate toward the wound site. Localized ROS/RNS generation affects FA dynamics by modulating focal adhesion proteins and remodeling the ECM. (**b**) Keratin 1 (*KRT1*) gene expression levels were determined via qPCR, and (**c**) keratin 1 (Krt1) protein expression levels were determined via WES analysis at both endpoints. (**d**) Immunofluorescence analysis on d9. (**e**) Quantification of cytokeratin (CK) in females (left) and males (right) on d9 and d20 in epidermal and dermal skin layers. (**f**) Representative picrosirius red (PSR)-stained connective ear tissue of female mice on days 9 and 20 in gas plasma-treated (lower panels) compared to untreated ear wounds and unwounded skin (upper panels), showing granulation tissue with collagen fibers (red) in fluorescence (left and middle images) and bright-field microscopy (right images). (**g-h**) The quantification of PSR in gas plasma-treated female (**g**) and male mice (**h**) compared to untreated and unwounded ear tissue, showing fiber intensity at d9 and d20 using ImageJ software. (**i**) The quantification of the fluorescence signal of the collagen (Col1A1) protein expression was shown in female and male mice at d9 and d20. (**j**) qPCR-based expression analysis of integrins **(*ITGB1, ITGA1/2/5/V6***), fibronectin (*FN1*), focal adhesion-related molecules, including focal adhesion kinase (*FAK*), vinculin (*VCL*), cadherin (*CDH1*), and vimentin (*VIM*) in female (left graphs) and male mice (right graphs) at both endpoints. **(k)** Representative WES images of Vcl, Vim, and Fak protein expression in female and male mice at d9 and d20. Gapdh expression served as an internal control. **(l)** Heat map of Fak, Vcl, and Vim protein expression in females (top three values) and males (bottom three values). **(m)** Immunostaining of Vcl (stars and arrows) in females at both time points. Data are expressed as mean ± S.D.; ∗p<0.05, ∗∗p<0.01, and ∗∗∗p<0.001 compared with untreated controls; scale bars are 50 μm (d, m), 200 μm (f); female and male mice were used on d9 and d20 (n > 4). D, dermis; ED, epidermis; FC, fold change**.**

Structural proteins present at focal adhesions (Fig. 5a) fulfill a variety of functions in the process of wound healing, encompassing roles such as cell migration, structural support, protection, and scar formation, thereby facilitating the efficient repair of damaged skin. The gene expression of keratin 1 (*KRT1*), which is found in keratinized structures of the epidermis, was significantly increased in females at d9 after gas plasma exposure. However, no significant changes were observed in males. A decrease was noted at later stages (d20) of wound healing in both sexes (Fig. 5b). Therefore, we examined the Krt1 protein expression by WES analysis, confirming qPCR levels (Fig. 5c), and distribution by antibody labeling of cytokeratins (CK), which are keratins of the epithelial skin structures. An augmented cytosolic expression was observed in the basal layers of the epidermis in both sexes following gas plasma exposure (Fig. 5d). The epidermal layers exhibited higher levels of CK expression in plasma-treated mice. However, the dermal staining corroborated the gene expression data and revealed significantly lower CK expression in females on days 9 and 20. There was also a trend toward reduced CK expression in the dermis of male mice (Fig. 5e). These data emphasize the dynamic regulation necessary for effective re-epithelialization at different stages, supporting the finding that the male response to gas plasma treatment may be more variable or less pronounced than the female response.

Fibroblasts proliferate and synthesize the extracellular matrix (ECM), including collagen and other structural proteins. Consequently, the subsequent phase of the study involved analyzing the potential impact of gas plasma on collagen fiber deposition. This analysis was conducted by employing Picrosirius Red (PSR) staining of wounds at both endpoint time points. Representative PSR-stained gas plasma-treated and untreated wounds highlighted the granulation tissue (arrows), including collagen fibers (red) in fluorescence (left and middle) and bright field (right) microscopy images (Fig. 5f). The connective ear tissue exhibited a high density of collagen fibers at d20 (bottom panel). In contrast, the epithelial layer was predominantly devoid of fibers, and immature, thinner collagen fibers (appearing as yellow or green fibers) were found at d9 (second and third panels). Furthermore, our observations revealed a predominance of mature, thicker collagen fibers, which manifested as bright red or orange fibers. In comparison with untreated wounds, a modest yet statistically significant increase in fiber deposition at 20 post-gas plasma exposure was observed in female (Fig. 5g) and at d9 in male mice (Fig. 5h). This finding provides substantial insights into the mechanisms underlying gas plasma-mediated tissue maturation and repair, which are associated with enhanced tissue integrity, strength, and stability following the healing process when compared with untreated wounds. PSR-stained fibers were significantly reduced in comparison to unwounded tissue at both endpoints, with the exception of gas plasma-treated female mice at d20. A comparison of collagen (Col) 1A1 protein expression levels in treated and untreated wounds revealed a tendency towards higher expression in the former (Fig. 5i). However, the beneficial gas plasma-mediated effects on cell structure were more pronounced in females than in males at the investigated time points.

Cell surface receptors, including integrins, function as critical mediators of interactions between cells and the ECM. These receptors facilitate bidirectional signal transmission, thereby regulating various cellular processes, such as migration, proliferation, and differentiation, which are crucial for wound healing [51]. Integrins, which heterodimerize to form functional transmembrane receptors, were differentially regulated in gas plasma-treated diabetic wounds depending on the stage of wound healing. The expression levels of the alpha-1 and V subunit of integrin (*ITGA1/V*) were found to be significantly increased (four to 16 times) in contrast to *ITGA5 and A2.* The heterodimer subunit of ITGA1 and A5, *ITGB1*, exhibited a downward trend at d20 in both sexes. Conversely, *ITGA6* demonstrated an upward trend after gas plasma treatment, with the exception of males at d20 (top graphs, Fig. 5j). The development of gas plasma-specific targeting of multiple integrins has the potential to provide novel treatment strategies for chronic wounds.

Focal adhesions serve as a mechanical anchor for cell migration and play an essential role in wound closure. Fibronectin 1 (FN1) has been demonstrated to form a provisional matrix that serves as a scaffold for the attachment and migration of fibroblasts and keratinocytes. This process is dynamically regulated. A substantial increase in *FN1* expression was observed, as quantified by qPCR analysis, which suggests that it may serve as a therapeutic target in cases of chronic wounds, which are often characterized by dysfunction. The activation of FAK in female mice indicated that the overexpression of FAK resulted in the formation of focal adhesions compared to males. Such focal adhesions are specialized structures that link the cells to the surrounding extracellular matrix. Furthermore, the analysis revealed that vinculin (*VCL*) mRNA expression showed a tendency to increase in all gas plasma-treated diabetic tissues. Concurrently, the level of E-cadherin (CDH1) was found to be elevated at both endpoints in both sexes, indicating a mesenchymal-epithelial transition (MET) that serves to reinstate the epithelial barrier. The topical application of gas plasma to the wound site promoted wound healing by enhancing MET, which in turn facilitates the restoration of epithelial characteristics and the reestablishment of cell-cell adhesions. This, in turn, enables the formation of new epithelial layers over the wound. Vimentin (*VIM*), a critical component of the cytoskeleton in myofibroblasts, was significantly upregulated in females at both endpoints. Yet, this phenomenon was not observed in males (lower graphs, Fig. 5j). This suggests a potential contribution to wound contraction through the exertion of contractile forces on the ECM. This process contributes to a reduction in wound area, thereby supporting the hypothesis of accelerated wound healing in females treated with gas plasma. The protein expression analysis of these targets exhibited a high degree of similarity to qPCR quantification, as evidenced by WES analysis for Fak, Vcl, and Vim (Fig. 5k–l) and by immunofluorescence microscopy for Vcl at both time points (Fig. 5m).

### 3.4 Gas plasma effects on restoration of barrier function in diabetic wounds

**Fig. 6:**
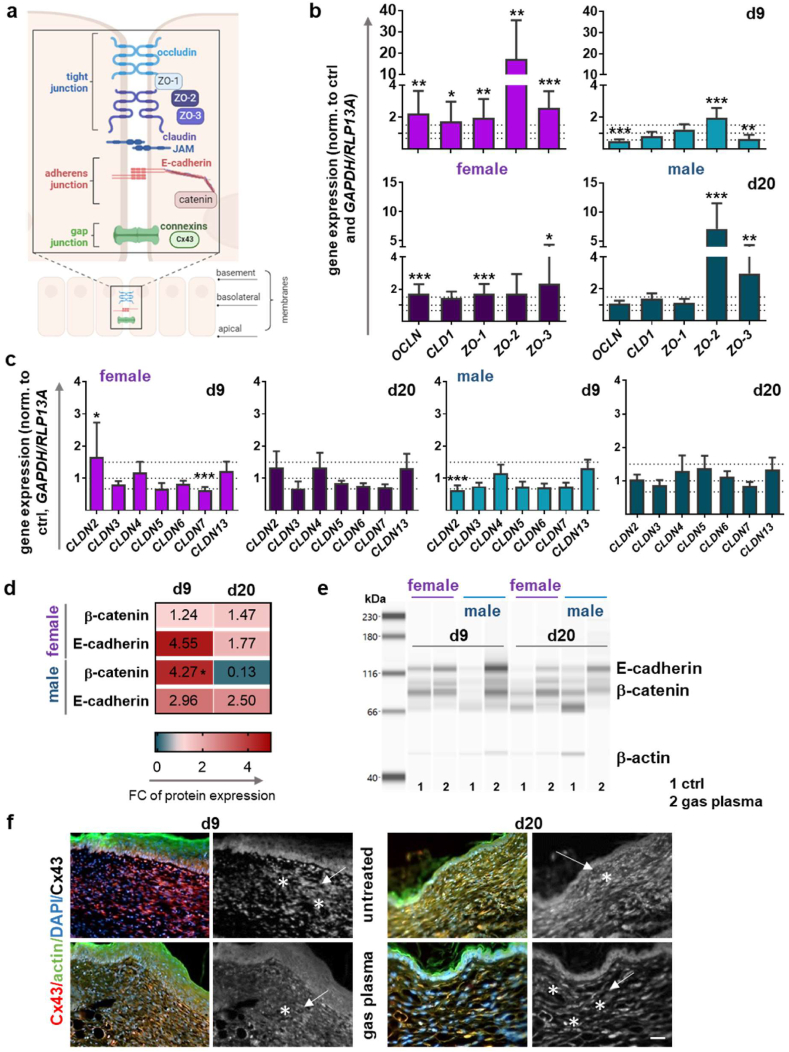
**Gas plasma treatment** modulates the **expression of junctional network targets** in diabetic wounds. (a) Overview of cell-cell connections, including tight junction (TJ), adherent junction (AJ), and gap junction (GJ). (b) Gene expression analysis of TJ **(*OCLN, CLDN1, ZO-1/2/3*)** was performed with total RNA isolated from the wound regions on both endpoints (d9/d20). **(c)** Gene expression analysis of claudins (*CLDN2/3/4/5/6/7/13*) was performed with total RNA isolated from the wound regions on both endpoints (d9/d20). **(d)** Heat map of β-catenin and E-cadherin protein expression in female (top two values) and male mice (bottom two values). **(e)** Representative WES image of β-catenin and E-cadherin protein expression (d9, d20) after gas plasma treatment (lanes 2) in female and male diabetic mice compared to untreated controls (lanes 1). β-actin was used as a reference. **(f)** Representative images of Cx43 (red and grey), actin (green), and DAPI (blue, nuclei) at d9 and d20 in female mice. Data are expressed as mean ± S.D.; ∗p<0.05, ∗∗p<0.01, and ∗∗∗p<0.001 compared with untreated controls; scale bar is 50 μm **(f)**, f**emale and male mice were used at d9 and d20 (n > 4)**. **FC, fold change;** CLDN, claudins; Cx43, connexin 43; JAM, junctional adhesion molecule; OCLN, occluding; ROS/RNS, reactive oxygen and nitrogen species; ZO-1/2/3, zona occludens proteins 1/2/3.

Tight junctions (TJ) and adherence junctions (AJ) play an essential role in wound healing by facilitating several processes central to tissue repair, regeneration, and barrier function (Fig. 6a). The mRNA levels of the TJ proteins occludin (*OCLN*) and claudin 1 (*CLD1*) were quantified, as they were found to be upregulated in females at both time points. This suggests that there is an apparent regulation of epithelial barrier permeability and maintenance of tissue integrity during the repair process. A significant (*OCLN*) and a tendency of lower expression (*CLDN1*) were detected at d9, along with a slight increase at d20, especially for *CLD1* in males. We observed an early and robust upregulation of several TJ proteins in females that could only be identified in males at later time points at d20. The zonula occludens proteins 1/2/3 (*ZO1/2/3*), which have been demonstrated to influence the rate and quality of re-epithelialization, exhibited significant upregulation at both endpoints, with the exception of d9 in males (Fig. 6b). ZO expression changes have the potential to influence immune cell migration and the overall tissue microenvironment at the wound site. The mRNA levels of other claudin family members were found to be differentially regulated and tended to exhibit analogous expression levels in females at both endpoints. However, only *CLDN2* was significantly upregulated, whereas *CLDN7* was significantly down-regulated on d9. In the male mice, claudins *CLD3/5/6/13* expression tended to decrease at d9 but increased at d20 (Fig. 6c). These results support the hypothesis that TJ proteins are present early on in the regenerating epidermis of diabetic wounds, thereby restoring the barrier function. Furthermore, the WES analysis revealed an increase in the expression levels of β-catenin and E-cadherin proteins. However, a robust downregulation of β-catenin at d20 was observed in the male mice (Fig. 6d–e), which may provide a molecular basis for the altered cell adhesion during the process of wound healing. Connexin 43 (Cx43), a prominent gap junction protein in the skin, frequently exhibits elevated levels during the initial phases of diabetic wound healing, potentially hindering the migration of crucial cells [52]. The topical application of gas plasma to diabetic wounds restored the balance of cell communication through the downregulation of Cx43 at d9, along with an increased expression at d20, as shown in representative images (Fig. 6f). This suggests a switch to a proper modulation of Cx43 expression during the various phases of wound healing.

### 3.5 Gas plasma-mediated changes in human wounds from diabetic patients

**Fig. 7:**
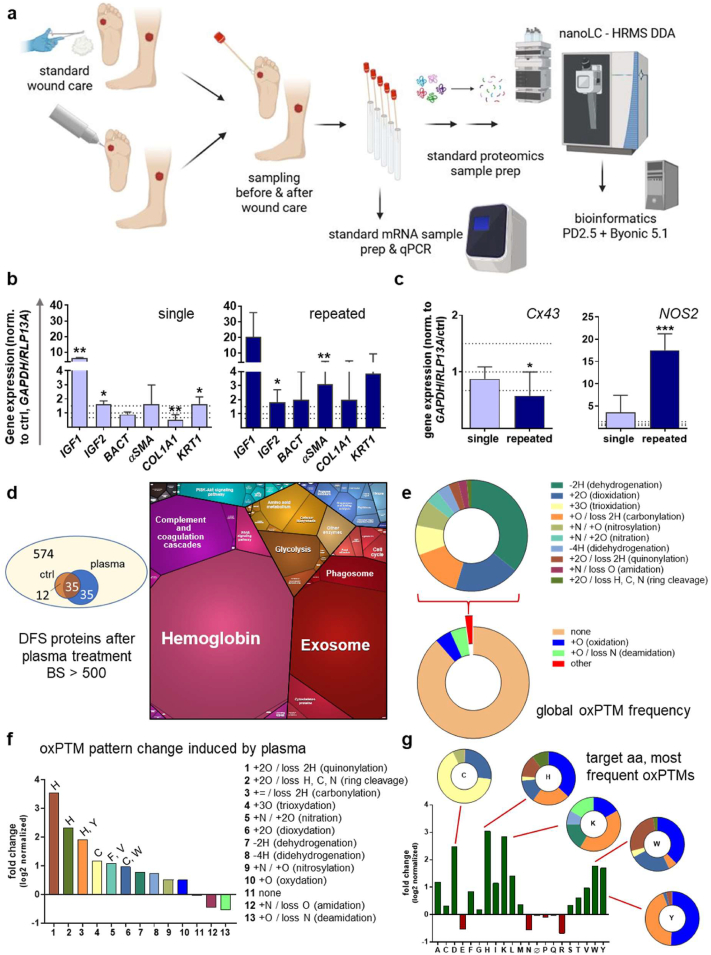
***In vi****vo* gas plasma treatment induced signaling pathways and protein oxidation in diabetic patients. **(a)** Swab-collected wound exudates after standard or gas plasma treatment and analysis with qPCR and mass spectrometry for oxidative protein modifications (oxPTMs). **(b–c)** The quantification of insulin growth factors 1 and 2 (*IGF1/2*), β-actin (*BACT*), smooth muscle actin (*αSMA*), collagen type 1A1 (*COL1A1*), keratin 1 (*KRT1*) **(b)**, connexin 43 (*Cx43*), and *NOS2***(c)** was conducted using qPCR. Data are presented as mean ± S.D.; ∗p<0.05, ∗∗p<0.01, ∗∗∗p< 0.001, as compared to controls (ctrl); n > 4. (d) A total of 574 proteins were found in the wound exudate of a DFS patient following gas plasma treatment, with 35 of these proteins being oxidized by gas plasma. Proteins identified reflect the presence of inflamed wounds with bleeding. **(e)** The total presence (or absence) of oxPTMs is shown to provide an overview of their frequency and the major types observed: The most prevalent modifications were oxidation (+O) and deamidation (+O/-N), with frequencies of 4.7 % and 4.6 %, respectively. Dehydrogenation (-2H) was the most prevalent rare oxPTM. (f) Gas plasma increased the presence of oxPTMs compared to controls (shown as FC of control). **(g**) The most prominent target amino acids were histidine, lysine, tyrosine, and tryptophan (shown as FC of control). Conceivable deamidation of N, Q, and R in the controls leads to an apparent reduction in the number of observed modifications in these amino acids after gas plasma treatment (see text). Approximately 89 % of the observed peptides did not bear an oxPTM (n = 10). BS; byonic score; DFS, diabetic foot syndrome; FC, fold change; NOS2, inducible nitric oxide; oxPTM, oxidative post-translational modifications; PD2.5, software version 2.5: prep, preparations; qPCR, quantitative polymerase chain reaction.

In the subsequent phase of the study, swabs collected from wound exudates following standard or gas plasma treatment were analyzed by qPCR and mass spectrometry to evaluate oxidative protein modifications. Wounds from diabetic patients were utilized for this analysis. The conductive mode of gas plasma was employed to augment the delivery of reactive signaling molecules, such as ROS/RNS, directly to the tissue (Fig. 7a). Following a brief incubation period (approximately 20 min), subsequent qPCR of diabetic wound exudates exhibited significant alterations in the gene expression profile between single and repeated treatments. Impaired expression of insulin-like growth factors (IGF) 1 and 2, along with their associated signaling pathways, has been identified as a contributing factor to delayed wound healing. This observation underscores the potential for therapeutic interventions aimed at enhancing wound repair in diabetic patients. Consequently, we analyzed the expression of *IGF1* and *IGF2* by qPCR*,* which revealed a substantial increase in gas plasma-induced expression under both conditions. However, it was only after repeated gas plasma treatment that a significant increase in expression levels of alpha-smooth muscle actin (*αSMA*) was observed, indicating the differentiation of fibroblasts into myofibroblasts, which are essential for wound contraction. Furthermore, a trend towards upregulation of collagen 1A1 (*COL1A1*) levels was identified (Fig. 7b), suggesting a contribution to the remodeling of the ECM by promoting collagen expression. Therapeutic strategies that target connexin 43 (Cx43) to either inhibit or modulate its activity hold promise for improving wound healing outcomes in diabetic patients, offering a potential solution to the chronic and non-healing nature of diabetic ulcers. In this study, we demonstrated a reduction of Cx43 expression even after repeated gas plasma treatment, in diabetic patients (left graph, Fig. 7c). These findings corroborate the preclinical results observed in a type 2 diabetic mouse model. In conditions requiring angiogenesis, such as wound healing, iNOS is often upregulated by inflammatory cytokines, including TNFα and IL1β, which promote angiogenic activity. Subsequent to undergoing gas plasma treatment, we confirmed significant upregulation of NOS2 levels, even after repeated gas plasma treatment (right graph, Fig. 7c).

At the protein level, 6845 have been identified across the patient samples and time points examined, with these identifications based on 4.4 million peptide spectra matches (PSMs). The proteins present in the samples reflected an inflamed wound with a diabetic background. The data set indicates a predominance of blood serum proteins, including albumin, hemoglobin, immunoglobulins, and proteins of the S100 family. In addition, structural proteins such as actin, keratins, myosin, and fibronectin are also present (Fig. 7d). A subsequent investigation employing overrepresentation analysis (PantherDB) showed that humoral immune response, complement activation, blood coagulation, and hydrogen peroxide-related processes were found to be significantly enriched. Of the proteins investigated in targeted mode using qPCR and WES analysis, only three were detected in proteomics: fibronectin, vinculin, and vimentin. These three proteins exhibited oxidative modifications (oxPTMs), including significant tryptophan modifications. However, their overall abundance was low. Therefore, we decided to include all available proteins collected from DFS patients in the search for oxPTMs. The presence of oxPTMs before and after the treatment in the proteins identified changed on a low but observable level. Among the 574 proteins evaluated at a specified time point following wound care and categorized by wound type (diabetic foot/leg ulcer), 70 proteins exhibited oxPTM after gas plasma treatment, as indicated by a high confidence interval (Byonic score >500). Standard wound care induced oxidations in 47 proteins, and the overlap between the two groups is 35 proteins (Fig. 7d). About 10 % of the PSMs were found to contain an oxPTM. The most prevalent modifications identified were deamidation of asparagine (-N/+O, *m*/*z* = +0.98) and oxidation of methionine (+O, *m*/*z* = +15.99), with each accounting for approximately 4.5 % of the modifications observed (Fig. 7e). While methionine oxidation was more prevalent after gas plasma treatment, asparagine deamidation appeared reduced. Deamidation of other amino acids increased moderately. Among the more minor oxPTMs observed in 2.3 % of the peptides, loss of hydrogen (-2H, dehydrogenation) and dioxidation (+2O) accounted for more than 50 % (Fig. 7e). A comparison of the numbers of oxPTMs before and after gas plasma treatment revealed a substantial increase in quinone formation (+2O/-2H), which increased nearly 12-fold. This was followed by ring cleavage (+2O, –H/C/N, 5-fold increase) and introduction of oxo groups (+O/-2H, carbonylation, 4-fold increase). The distribution of these oxPTMs was predominantly observed in histidine and tyrosine residues (Fig. 7f). We observed minor nitration, with an average of two to seven peptide spectrum matches per sample (<0.01 %), primarily at valine (V) and phenylalanine (F). Trioxidation (+3O) was observed almost exclusively in cysteine residues (formation of a sulfonic acid group). Irrespective of individual oxidative modifications, amino acids most often attacked by the gas plasma were histidine, tryptophan, tyrosine, cysteine, and lysine (Fig. 7g).

## 4 Discussion

Type 2 diabetes mellitus (T2DM) is a chronic metabolic disorder characterized by insulin resistance and impaired insulin secretion. This condition leads to several complications, including hyperglycemia and delayed wound healing. These complications arise due to impaired angiogenesis, reduced immune response, and persistent inflammation [6]. These wound-healing defects can lead to an increased risk of chronic ulceration and infection, which pose significant challenges for treatment. The exposure of biological surfaces to gas plasma has emerged as a promising therapeutic modality to improve wound healing [53]. Cold physical plasma has been demonstrated to generate reactive oxygen and nitrogen species (ROS/RNS) [54], which have been shown to modulate cellular signaling pathways, thereby accelerating tissue regeneration, promoting angiogenesis, and reducing microbial load [55,56]. This association establishes a link between redox biology and specific clinical effects and positions gas plasma treatment as a novel tool to address the wound-healing deficits associated with T2DM. A substantial body of research has employed preclinical rodent models to examine the effects of gas plasma-induced healing (reviewed in Ref. [53]), including a modest number of studies on T2DM mice [[57], [58], [59], [60]]. Nevertheless, the utilization of mouse models remains indispensable in the realm of T2DM-related wound healing research and the evaluation of gas plasma-mediated interventions. Diabetic mouse models, such as those induced by a high-fat diet, exhibit metabolic and wound-healing profiles analogous to human T2DM [32,[61], [62], [63], [64], [65], [66], [67]]. In this study, an obese mouse model of T2DM (B6.Cg-Lepob/J) was utilized as a controlled platform to investigate the mechanisms underlying delayed healing and to evaluate the efficacy of gas plasma treatment. Finally, high-resolution mass spectrometry of wound fluids collected from diabetic patients provided valuable insight into the wound healing process and oxidative post-translational modifications.

ROS/RNS are naturally occurring molecules that are produced during cellular metabolism [68]. It has been established that ROS/RNS play essential roles in cellular signaling and defense mechanisms at physiological levels. However, oxidative stress in diabetic wounds has been demonstrated to impede critical healing stages, including the resolution of inflammation, angiogenesis, and collagen synthesis [69]. The persistent state of elevated blood glucose levels and the body's inability to effectively utilize insulin, characteristics of T2DM, give rise to an overproduction of ROS/RNS. This, in turn, leads to a compromised antioxidant defense system, thereby inducing a state of oxidative stress [70]. This oxidative stress has been shown to damage cellular structures, including lipids, proteins, and DNA, and to disrupt key processes involved in wound healing. Maintaining a balance between antioxidant and pro-oxidant processes is imperative for regulating ROS/RNS production and creating an optimal wound environment that supports the multiple stages of cellular and molecular wound healing processes [18,71]. It can be assumed that the activity of antioxidant enzymes is modulated by gas plasma-derived ROS/RNS [72,73]. Increased ROS/RNS has been shown to trigger intracellular responses, including the activation of the nuclear factor erythroid 2-related factor 2 (Nrf2) signaling pathway in diabetic mouse models and human wound samples [39,47,74]. However, the transition from the inflammatory to the proliferative phase is a critical step in the healing process, and mounting evidence suggests that a compromised transition is associated with chronic wounds. Thus, targeting factors that impact this phase transition (e.g., tight control of redox signals) may be a rational approach to therapeutic development [75]. We hypothesized that short external bursts of ROS/RNS can help “reset” or reprogram local cellular antioxidant responses and redox-sensitive signaling pathways. This improves cellular function and promotes healing despite chronic endogenous oxidative stress. ROS/RNS also play a critical role in redox-chemical pathways, such as inflammation [76]. As secondary messengers for immune cells, they orchestrate healing-related responses and play a role in host defense, exhibiting bacteriostatic effects in the wound environment [77,78]. We identified a gas plasma-promoted expression of antimicrobial peptides, including defensins and lysozymes, which was mainly observed in females, confirming the bacteriostatic effect of gas plasma [55,79,80]. Given the documented efficiency of gas plasma in inactivating a broad spectrum of microorganisms, the application of this technology in the treatment of T2DM patients with severe bacterial infections in chronic wounds is a rational approach. Remarkably, the oxidation of nucleic and organic acids induced by gas plasma can be excluded for RNA and DNA that are enclosed by the cell membrane. A series of experiments was conducted using standard models to ascertain the genotoxicity of the subject material and reveal any genetic instability [81,82]. Robust protective mechanisms offered by other biomolecules effectively impede direct assaults on RNA/DNA. On the contrary, previous research in plasma medicine has shown that gas plasma-derived species can targeted isolated nucleic acids [83]. Certain enzymes, such as myeloperoxidase [84], and tyrosine kinases [85], may undergo oxidation during treatment, which could reduce or modulate their activity. Furthermore, the application of intense gas plasma treatment has been shown to inhibit phospholipase A2, a pivotal enzyme in lipid metabolism [10]. A substantial body of literature shows that lipids can be targeted by gas plasma-derived species [[86], [87], [88], [89]]. Under suitable conditions, fatty acid side-chain oxidations were observed, resulting in membrane instability and increased permeability. However, even under the selected model conditions, the treatment times required to achieve this, are considerably longer than those necessary for non-enzymatic oxidative post-translational modifications (oxPTMs) in proteins/peptides [44,87,90]. Consequently, it is hypothesized that lipid oxidation will play a minor role in diabetic wounds.

The application of gas plasma has been demonstrated to enhance neovascularization [39], which is also supported by the fact of increased expression of inducible nitric oxide synthase (iNOS) [13]. In contrast, high levels of ROS/RNS perpetuate chronic inflammation and prevent the formation of functional blood vessels, which are essential for delivering oxygen and nutrients to the wound site. Tivita's hyperspectral imaging technology facilitates the non-invasive, real-time observation of microcirculatory parameters [91]. Our study offers multiple lines of evidence that exposing diabetic wounds to gas plasma reduces tissue water content in gas plasma-treated diabetic wounds, indicating improved healing through re-epithelialization and lymphatic drainage [[91], [92], [93]]. The decline in near-infrared (NIR) reflectance in hyperspectral imaging measurements can be attributed to various factors related to the skin's optical properties [94]. We observed that a higher melanin content leads to a reduction in NIR reflectance [95] as compared to the light-skinned nude mouse model [17,42,96]. In the female mouse model, oxygen saturation levels increased earlier (d0, d9-12) than in males (d16), underscoring the disparities between the sexes. However, the hypothesis that an early treatment for females and a late treatment for males would be optimal is speculative. Due to the experimental design, it is not possible to conclude whether a later onset of gas plasma therapy in male mice would have a similar or even better effect than in females. Further studies are necessary to determine the impact of varying therapy initiation times. However, the temporal response to gas plasma therapy is influenced by sex-specific wound healing dynamics. It has been established that there are gender-related disparities in hormonal regulation, immune function, oxygenation, angiogenesis, and response to oxidative stress in mice [97]. Females generally exhibit a more rapid initial inflammatory response and may transition more rapidly to the proliferative phase of wound healing [47]. This phenomenon is attributable to estrogen's beneficial effects on angiogenesis and re-epithelialization [98]. Testosterone has been demonstrated to influence males, causing a protracted transition from inflammation to proliferation and resulting in delayed neovascularization [99]. Subsequent gas plasma therapy in males may align more closely with their delayed transition into the proliferation and neovascularization phases, thereby maximizing the effect on oxygen delivery and tissue repair. It is hypothesized that early interventions in wound treatment are essential for females to maximize the therapeutic benefits of gas plasma. At the same time, a later application may be more appropriate for males due to their unique healing processes. Overall, the reduced number of significant processes and the reduced expression of genes and/or proteins at the d20 compared to d9 likely reflects the natural progression of healing in both sexes. The early stages involve robust molecular activity related to inflammation and tissue remodeling. As healing progresses, the biological focus shifts toward tissue maturation and homeostasis, resulting in fewer differentially expressed or active molecules.

Physical barriers are known to provide innate protection, with examples including skin cell overlaps and chemical barriers [80]. Changes in the expression and localization of integrins and tight junction proteins, such as occludin and claudins, have been observed to occur during inflammatory responses and tissue repair [100]. These alterations could potentially affect cell migration, immune cell trafficking, and the overall inflammatory milieu at the wound site. Our findings corroborate the initial observations of tight junction proteins in the regenerating epidermis of diabetic wounds, as also reported in previous studies [101,102]. Other structural proteins, such as keratins, have been shown to modulate the inflammatory response during wound healing. These proteins can influence the production of various signaling molecules and cytokines, thereby helping to regulate the immune response at the wound site [103]. During this phase, epidermal remodeling triggers a shift in keratin isoforms, accompanied by the downregulation of keratins. T2DM is characterized by a nitric oxide (NO)-deficient state and a severely impaired inflammation [104]. As mentioned above, we also identified a significant, albeit transient, increase in inducible NO synthase expression in mouse and human wounds, resulting in alterations to the deposition of the extracellular matrix (ECM). Pathophysiologically, dysregulated tissue repair in diabetes has been linked to an imbalance in ECM deposition in the cellular compartments in the dermis [105]. Collagen, a major ECM component, facilitates the process of tissue repair by providing structural support to tissues [106]. Augmenting collagen synthesis has been shown to form more robust, organized tissue [107], as demonstrated by picrosirius red staining after gas plasma treatment [[108], [109], [110]]. This process leads to the formation of granulation tissue, which provides a structural framework for normal tissue regeneration [111]. Integrins play a critical role in connecting the cell cytoskeleton to the ECM, thereby facilitating cellular processes such as cell adhesion, migration, proliferation, and the formation of ECM [112]. Gas plasma treatment significantly affected cell adhesion by regulating focal adhesion kinase (Fak), which is vital in maintaining skin barrier function [17,113]. Integrin-mediated signaling pathways (e.g., Fak) can modulate the expression of gap junction proteins or their assembly, thereby impacting intercellular communication. The targeted expression of connexin 43 (Cx43), the most prevalent gap junction protein in rodent skin, has the potential to improve diabetic wound healing. In diabetic rats, keratinocyte migration does not begin until Cx43 is downregulated [114]. We have demonstrated that this phenomenon occurs after gas plasma treatment in our mouse model and in diabetic exudates from patients.

PCNA expression is often used as a marker to identify cells actively dividing and synthesizing DNA, which is characteristic of cells in the proliferative phase. As these cells proliferate and synthesize the ECM, including fibrillary collagens and other structural proteins, they become PCNA-positive. In the late stages of wound healing, apoptosis plays a critical role in several aspects of tissue repair. In diabetic wounds, apoptosis occurs prematurely and over a prolonged period. This premature apoptosis kills keratinocytes, fibroblasts, and endothelial cells, which impairs re-epithelialization, angiogenesis, and granulation tissue formation. It also sustains inflammation and delays wound closure. Therefore, dysregulated apoptosis is a key driver of chronicity [115]. The effect on the p53 signaling pathway was less than expected during the examined time periods. We consider this a therapeutic success because it suggests that plasma therapy counteracts the harmful effects of dysregulated apoptosis on diabetic wound healing. However, since no additional time points were analyzed, it is not possible to draw conclusions about the temporal dynamics of apoptosis. Additionally, gas plasma treatment modifies the mechanical properties of the ECM, which in turn can impact the mechanotransduction pathways that regulate the activity of yes-associated protein (YAP) and transcriptional coactivator with PDZ-binding motif (TAZ) [116]. YAP/TAZ activity is essential for skin homeostasis and cell proliferation during wound repair [117]. The Hippo pathway regulates endothelial cell proliferation, migration, and survival through the activity of its effectors, YAP/TAZ. These effects subsequently impact vascular sprouting, vascular barrier formation, and vascular remodeling [118]. The current study suggests an association between the Hippo transcription factor activation and improved wound healing, as previously proposed [26]. We observed YAP (TAZ) activation after plasma treatment, both *in vivo* on day 9 and *in vitro* after 24 h. However, it could no longer be regulated, either permanently or temporarily, because the molecular environment changed during diabetic wound healing. We also found that the expression of CTGF and CYR61 did not depend solely on YAP/TAZ activation, both *in vitro* and *in vivo*. YAP/TAZ are essential regulators of Hippo signaling, while CTGF and CYR61 are multi-pathway integrators that respond to the Hippo pathway, as well as the TGFβ [119], Wnt/β-catenin [120], mechanical, and integrin/FAK-mediated signaling pathways [121].

In recent years, wound exudates, which are highly dynamic body fluids, have come into focus for their potential diagnostic value. A standardized sampling procedure combined with dedicated preparation of wound fluids collected from diabetic patients provided valuable insight into wound processes. Due to the short interval between gas plasma treatment and sampling, no changes in protein expression were anticipated. Air drying and the removal of protein mass during wound care reduce the amount of sample that can be collected noninvasively. This yields a lower number of identified proteins compared to sampling prior to iatrogenic intervention. The observed proteome was dominated by proteins induced by bleeding during wound care; however, wound-related proteins such as myeloperoxidase [122], Parkinson's disease protein 7 [123], and matrix proteins were also detected. Approximately 12 % of the proteins exhibited oxPTMs, some of which were background modifications (e.g., methionine oxidation and asparagine deamidation). Asparagine deamidation is connected to inflammation and protein aging, acting as a biological clock that controls protein recycling [124]. This oxPTM was found to be less prevelant after gas plasma treatment, which may reflect either increased bleeding due to local hyperperfusion associated with gas plasma-induced stimulation or a bioinformatics effect. To minimize computational costs, only one modification per peptide was permitted. Introducing any additional oxPTMs would exclude peptides with asparagine deamidation from the analysis. This effect is observed with the amino acids glutamine (Q) and arginine (R). Signs of oxidative events triggered by the gas plasma were found in the electron-rich aromatic or heteroaromatic amino acids, as well as in lysine. Simultaneously, aromatic amino acids form quinones, keto-enol tautomers (histidine), or ring-open structures; the ε-amino group of lysine forms chloramines, which yield deamidated products. The relevant gas plasma-derived reactive species are atomic oxygen (O), hydroxyl radicals (HO∗), singlet oxygen (^1^O_2_), and hydrogen peroxide (H_2_O_2_). Contrary to expectations from model experiments, tyrosine nitration was not observed [45,125]. The relevant oxidative intermediate, peroxynitrite (ONOO^−^), either does not form in the wound or forms in such small amounts that the number of nitrated tyrosine molecules remains too low for detection without enrichment. Currently, it is not possible to provide direct evidence that these oxPTMs contribute to faster wound closure in diabetic wounds. This would require the ability to manipulate the major proteins in the wound fluid by introducing oxidized amino acids with the respective oxPTMs. However, most amino acids are not available in an oxidized form. Furthermore, it is impossible to incorporate a molecule into cellular proteins by manipulating the ribosomal protein synthesis. One possible approach is to use siRNA to silence the production of distinct proteins, but this method is limited by the fact that a mixture of proteins is oxidized. Perhaps, only the interplay of these proteins affects healing, not a single knockdown. Furthermore, at the time of treatment, proteins have already been oxidized by gas plasma-derived reactive species. Changes in protein turnover, processing by antigen-presenting cells (APCs), protein-protein interaction, and remaining functionality downstream may arise, precluding thorough investigation in a clinical setting. Accordingly, only results from model scenarios can be consulted: (i) enzymes are inactivated by (intense) gas plasma treatment [10,85], (ii) peptides change their chemical composition and physiological signaling [45,126], (III) alterations of immunogenicity [11]. In these individual cases created to study the chemistry of amino acid, peptide, or protein modification by gas plasma-generated species, non-enzymatic oxPTMs were introduced, thereby altering the activity of the respective biomolecules. In our study, identical oxPTMs were found in the wound fluids of diabetic patients observed after gas plasma treatment.

Currently, there is no linear relationship between the observed non-enzymatic oxPTMs that are attributed to the interaction between gas plasma and the proteins in the wound fluid. Current knowledge suggests that the activation of these pathways is not exclusive to diabetic wound healing, but rather represents a more general response to tissue injury [17,36,96,127,128]. However, the amplitude, regulation, and consequences of this activation can be profoundly altered by the diabetic environment. Further research is necessary to show their specificity to diabetic wound healing. Additionally, there is very little data indicating the presence of oxPTMs in human or animal wound fluids. This is due to the experimental (and ethical) hurdles that are difficult to overcome. Detecting oxPTMs in a complete wound proteome is technically challenging and requires advanced bioinformatics. Our research focuses on non-enzymatic oxPTMs to investigate the underlying chemistry of gas plasmas. As a result, we have built a database and acquired prior knowledge that facilitates the mapping of oxPTMs in complex samples [44,45,129,130]. Thus far, no enrichment strategies have been developed for oxidized proteins or peptides following enzymatic digestion other than for cysteine modifications and tyrosine phosphorylation. Consequently, oxPTMs are scattered among the data of non-modified proteins and peptides. The lack of reference compounds further complicates analytical approaches. Due to this situation, few studies have been published, making this study a pioneer in the field. In summary, the wound proteins exhibited site-directed oxidative modifications that can be attributed to the gas plasma treatment of diabetic human wounds. As demonstrated for model proteins, these modifications can either lead to changes in the biological activity [10,84,85] or increased recognition by the immune system [11] according to downstream events, ultimately contributing to the observed effects of gas plasma *in vivo*.

Several limitations and challenges of the present study must be acknowledged. First, the study design prevented the use of other oxygen imaging techniques. Using a single method can lead to method-specific biases and limited spatial and temporal resolution, as well as prevent cross-validation. Absolute quantification and insights into the resolution of fine-scale heterogeneity in plasma-induced tissue oxygenation require complementary techniques, such as EPR spectroscopy, oxygen microelectrodes, or magnetic resonance microscopy/positron electron microscopy (MRI/PET). Future patient studies could incorporate different oxygen measurement methods to validate the current results and increase confidence in the accuracy of the oxygen data. Secondly, wound healing was only evaluated at two endpoints: on days 9 and 20. While these time points cover the middle and late phases of tissue repair, they do not allow for an evaluation of the initial stages, which could be essential for understanding the pathophysiology of diabetic wounds. Processes such as inflammatory resolution, early apoptotic events, and rapid activation or suppression of intracellular signaling cascades commonly occur within the first hours to days after injury. Because sampling was limited to days 9 and 20, temporary but biologically significant changes may have been overlooked. Consequently, interpretations about mechanisms that depend on early timing (e.g., the initiation of apoptosis or reparative signaling) remain speculative. Third, our *in vivo* data do not provide information about the timing of YAP activation or the regulation of its downstream targets during diabetic wound healing in relation to diabetes mellitus (DM). To address this gap, we conducted *in vitro* experiments to demonstrate plasma-induced YAP activation under controlled conditions. However, *in vitro* systems cannot fully replicate the complex cellular interactions or the temporal resolution of signal transduction that occur in diabetic wounds. Therefore, extrapolating *in vitro* results to the *in vivo* environment of diabetic wounds should be done with caution. Taken together, these limitations highlight the need for more detailed temporal mapping of wound biology in diabetic patients. Future studies should include earlier and more frequent sampling to capture the dynamics of apoptosis and rapid signaling changes. For example, sampling could be performed during the first three days. Additionally, *in vivo* mechanistic experiments using YAP/TAZ inhibition in wild-type mice are necessary to validate *in vitro* observations and to confirm whether YAP/TAZ is indeed required for the healing effects to establish causality. To analyze the mechanistic role of YAP/TAZ in gas plasma-induced wound healing, knockout mice are a robust and unambiguous model because they eliminate many of the potential confounding factors inherent in pharmacological or siRNA approaches. Although knockout models are complex and require significant resources, future investigations will utilize them. Closing these gaps will improve our understanding of the early molecular processes involved in wound healing in diabetes and strengthen the study's translational relevance. Furthermore, translating preclinical biomedical research findings into clinical applications in humans poses a significant challenge. Adjusting gas plasma treatment parameters, including duration, intensity, and frequency, may be necessary to optimize results and personalize treatment in clinical settings, accounting for the different characteristics of female and male patients with diabetes.

## 5 Conclusion

This study highlights the molecular consequences of gas plasma treatment in preclinical wound models, encompassing those associated with diabetes. Firstly, this study elucidates the beneficial role of gas plasma-enhanced wound healing. Secondly, the utilization of hyperspectral imaging to assess four microcirculatory parameters revealed alterations in tissue oxygenation, perfusion, hemoglobin levels, and water content. These alterations may be linked to gas plasma-induced modifications in tissue structure, including changes in collagen content and extracellular matrix (ECM) composition. Thirdly, evidence was presented demonstrating that gas plasma activates the Hippo pathway effector, YAP, which regulates pivotal processes in wound healing, including angiogenesis. Finally, we confirmed preclinical observations of gas plasma-induced changes in junctional target expression (e.g., decreased Cx43 levels) and increased angiogenic factor expression (e.g., NOS2 expression) in diabetic patients. Wound proteins exhibited site-directed oxidative post-translational modifications that can be attributed to gas plasma treatment of human diabetic wounds. This knowledge is critical for translating innovative therapies for treating diabetic wounds into clinical applications.

## Funding

This work was supported by the German 10.13039/501100002347Federal Ministry of Education and Research (BMBF; now: German Federal Ministry of Research, Technology, and Space, BMFTR), grant numbers 03Z22DN11 (to SB), 03Z22Di1 (to SB), and 03Z22DN12(to KW). The funder had no role in study design, data collection, data analysis, manuscript preparation, and/or publication decisions.

## CRediT authorship contribution statement

**Anke Schmidt:** Conceptualization, Data curation, Formal analysis, Investigation, Methodology, Project administration, Software, Supervision, Validation, Visualization, Writing – original draft, Writing – review & editing. **Kristian Wende:** Data curation, Formal analysis, Investigation, Methodology, Resources, Software, Validation, Visualization, Writing – review & editing. **Liane Kantz:** Investigation, Writing – review & editing. **Thomas von Woedtke:** Funding acquisition, Writing – review & editing. **Sander Bekeschus:** Funding acquisition, Methodology, Project administration, Resources, Software, Writing – review & editing.

## Declaration of competing interest

The authors declare no conflict of interest.

## Data Availability

Data will be made available on request.
