# Supplementary material for: Gas plasma therapy of murine and human diabetic wounds is associated with junctional and hippo signaling and oxidative protein modifications
Source: Redox Biol. 2025 Nov 28;89:103951. doi: 10.1016/j.redox.2025.103951 (PMC12720129; doi:10.1016/j.redox.2025.103951)
Supplement: Multimedia component 1 [file mmc1.pdf]

Gas plasma therapy of murine and human diabetic wounds is associated with junctional and hippo signaling and oxidative protein modifications

Anke Schmidt[1], Kristian Wende[1], Liane Kantz[1], Thomas von Woedtke[1,2], *Sander Bekeschus[1,3]

[1] ZIK *plasmatis*, Leibniz Institute for Plasma Science and Technology (INP), Felix-Hausdorff-Str. 2, 17489 Greifswald, Germany

[2] Institute of Hygiene and Environmental Medicine, Greifswald University Medical Center, Sauerbruchstr., 17475 Greifswald, Germany

[3] Department of Dermatology, Venerology, and Allergology, Rostock University Medical Center, Strempelstr. 13, 18057 Rostock, Germany

***Correspondence**: sander.bekeschus@med.uni-rostock.de

Keywords: CAP; diabetes mellitus; Hippo signaling; hyperspectral imaging; human wound exudates; mass spectrometry; plasma medicine; reactive species; ROS/RNS

Running head: Gas plasma-induced wound healing signaling in T2DM

Abbreviations: aj, adherence junctions; AMP, antimicrobial peptides; Cx, connexin; gj, gap junctions; ECM, extracellular matrix; HSI, hyperspectral imaging; oxPTM, oxidative post-translational protein modificatios; T2DM, type 2 diabetes mellitus; TJ, tight junctions; ROS/RNS, reactive oxygen species

Supplemental data

Table A1. Clinical characteristics of patient samples. qPCR, quantitative polymerase chain reaction (n ≥ 4); HMRS, high resolution mass spectrometry (n = 10).

Table A2. Murine gene-specific primers used in qPCR.

Table A3. Human gene-specific primers used in qPCR.

Table A4. Oxidative protein modifications searched by the ProteomeDiscoverer node Byonic based on [1-4]. DFS, diabetic foot syndrome; oxPTMs, oxidative protein modifications; m, man; w, women (n = 10).

Table A5. Gas plasma-treated DFS wounds and oxidized proteins exclusively identified. Number represents the frequency of observed peptide spectra matches with Byonic score > 500 carrying an oxidative modification. % Ox (oxidation) indicates how many observations contain an oxidative modification (n = 10). DFS, diabetic foot syndrome.

Figure A1. Re-epithelialization after gas plasma treatment in diabetic mice. The rate of wound closure is plotted as the percentage reduction of the original wound area over time for treated mice when compared to the untreated wounds in females (**a**) and males (**b**) at d9 and d20 (n ≥ 4). (**c**) Hyperspectral images of NIR for the untreated control (left image) and a gas plasma-treated wound (middle and right images) at d9. (**d**) Quantification of NIR values over time (d0-d20) in females. (**e**) Hyperspectral images of NIR for the untreated control (left) and a gas plasma-treated wound (middle and right images) at d0 (top panel) and d9 (bottom panel). (**f**) Quantification of NIR values over time (d0-d20) in males. Points of interest were placed on the wound area (red circle and arrows in c, e). The color scale ranges from blue (0) to red (100%). Days of interest were shown as indicated (purple arrow in d, f). Data are presented as the mean ±S.D.; *p<0.05, and **p<0.01, ***p<0.001 compared to untreated controls. The total number of measurements collected was 748 (n ≥ 4). d, days; NIR, near-infrared index; w/o, without wounding.

Figure A2. NOS2 and antimicrobial peptide expression. (**a**) qPCR-based expression analysis of nitric oxide synthase 2 (*NOS2*, inducible) at both endpoints. (**b**) Immunofluorescence microscopy of iNOS. (**c**) Wounds of diabetic mice were either exposed to gas plasma (10 s) or left untreated (ctrl). (**d**) Gene expression analysis of antimicrobial peptides (AMPs): defensins (*DEFB1/6/26/28/50*) and lysozymes (*LYZ1/2*) using total RNA isolated from the wound regions at both endpoints (d9/d20). Graphs show mean ± standard error of the mean (SEM). *p<0.05, and ***p<0.001 compared with untreated controls; females and males were used on d9 and d20 (n ≥ 4).

Figure A3. Gas plasma treatment modulates protein expression in diabetic wounds. (**a**) Representative WES image of YAP, β-actin, and Gapdh protein expression (d20) after gas plasma treatment in female and male diabetic mice compared to untreated controls (ctrl). (**b**) Representative WES image of pYAP (Ser-127) and TAZ protein expression (d9) after gas plasma treatment in female and male diabetic mice compared to untreated controls (ctrl). β-actin and Gapdh were used as references (n≥4). (**c**) qPCR-based expression analysis of TAZ, CTGF, and Cyr61 6 and 24 h after plasma treatment in skin cells. TAZ, transcriptional coactivator with PDZ binding motif; YAP, yes-associated protein; pYAP, phosphorylated form of YAP.

Appendix

Table A1

diabetic patient	sex	age	wound type	~size of wound (cm^2)	plasma treatment	analysis
1	m	76	ulcus (DFS)	136	single	qPCR
2	m	86	ulcus (DFS)	159	single	qPCR
3	m	82	ulcus (DFS)	1	single	qPCR
4	m	59	unknown	3	single	qPCR
5	m	84	ulcus mixtum	8	single	qPCR
5	m	76	ulcus (DFS)	136	repeated	qPCR
6	m	86	ulcus (DFS)	159	repeated	qPCR
7	w	89	ulcera crurus	12	repeated	qPCR
8	m	78	deep ulcus	9	repeated	qPCR
9	m	62	ulcus (DFS)	4.0	single	HRMS
10	m	88	ulcus (DFS)	23	single	HRMS
11	m	66	ulcus (DFS)	4.4	single	HRMS
12	m	79	ulcus (DFS)	1.8	single	HRMS
13	m	79	ulcus (DFS)	7.8	single	HRMS
14	w	56	ulcus (DFS)	1.2	single	HRMS
15	m	74	ulcus (DFS)	3.2	single	HRMS
16	w	59	ulcus (DFS)	4.3	single	HRMS
17	m	44	ulcus (DFS)	3.0	single	HRMS
18	m	66	ulcus (DFS)	3.4	single	HRMS

Table A2

gene name	gene ID	primer sequences (3`- 5`)
colony-stimulating growth factor	*CTGF*	GAC CCA ACT ATG ATG CGA GCC<br>CCC ATC CCA CAG GTC TTA GAA C
cysteine-rich angiogenic inducer 61	*CYR61*	TAA GGT CTG CGC TAA ACA ACT C<br>CAG ATC CCT TTC AGA GCG GT
tumor suppressor protein 53	*p53*	AAA GGA TGC CCA TGC TAC AGA GGA<br>AGG ATT GTG TCT CAG CCC TGA AGT
Bcl-2-associated X protein	*BAX*	ACA GCA ATA TGG AGC TGC AGA GGA<br>TGT CCA GCC CAT GAT GGT TCT GAT
cyclin-dependent kinase inhibitor 1	*CDKN1A*	GGA ATT GGA GTC AGG CGC AGA T<br>GAA GAG ACA ACG GCA CAC TTT GCT
hypoxia-inducible factor 1 alpha	*HIF1A*	GGG GAG GAC GAT GAA CAT CAA<br>GGG TGG TTT CTT GTA CCC ACA
integrin A1	*ITGA1*	GAC AGC CCT TGG AAT AGA CAC<br>GTT GTC ATG CGA TTC TCC ATC A
integrin A2	*ITGA2*	TGT CTG GCG TAT AAT GTT GGC<br>TGC TGT ACT GAA TAC CCA AAC TG
integrin A5	*ITGA5*	TGC AGT GGT TCG GAG CAA C<br>TTT TCT GTG CGC CAG CTA TAC
integrin AV	*ITGAV*	AAA GAC CGT TGA GTA TGC TCC A<br>ATG CTG AAT CCT CCT TGA CAA AA
integrin A6	*ITGA6*	GGG ATC GTC CGT GTA GAA CAA<br>TCT CTC CAC CAA CTT CAT AGG G
integrin B1	*ITGB1*	TGG TCA GCA ACG CAT ATC TGG<br>GAT CCA CAA ACC GCA ACCT
focal adhesion kinase	*FAK*	GAG TAC GTC CCT ATG GTG AAG G<br>CTC GAT CTC TCG ATG AGT GCT
vinculin	*VCL*	GCT TCA GTC AGA CCC ATA CTC G<br>AGT AAG GGT CTG ACT GAA GCA T
vimentin	*VIM*	CGT CCA CAC GCA CCT ACA G<br>GGG GGA TGA GGA ATA GAG GCT
fibronectin	*FN1*	AGG AAG CCG AGG TTT TAA CTG<br>AGG ACG CTC ATA AGT GTC ACC
keratin 1	*KRT1*	CTC AGT ATA TAA GGG CAC GGC ACT<br>GAC TCA TGA TGC CTT AGA GAG AGG T
E-cadherin	*CDH1*	CAC CTG GAG AGA GGC CAT GT<br>TGG GAA ACA TGA GCA GCT CT
occludin	*OCLN*	TGT GGG ATA AGG AAC ACA TTT ATG A<br>CAG ACA CAT TTT TAA CCC ACT CTT CA
claudin 1	*CLDN1*	TGC CCC AGT GGA AGAT TTA CT<br>CTT TGC GAA ACG CAG GAC AT
claudin 2	*CLDN2*	ATG CCT GGA AGC CAG AAG TC<br>CCC GCA ATA AAC AAG AAG GGT AA
claudin 3	*CLDN3*	ACC AAC TGC GTA CAA GAC GAG<br>CGG GCA CCA ACG GGT TAT AG
claudin 4	*CLDN4*	ATG GCG TCT ATG GGA CTA CAG<br>GAG CGC ACA ACT CAG GAT G
claudin 5	*CLDN5*	GCA AGG TGT ATG AAT CTG TGC T<br>GTC AAG GTA ACA AAG AGT GCC A
claudin 6	*CLDN6*	TGC AAG GTG TAT GAC TCA CTG T<br>GAC GAG ACT TGG AGT TCC TAT CT
claudin 7	*CLDN7*	CTG GAG GCA TTG TTT TCA TTG TG<br>CAT GGG CGT CAA GGG GTT A
claudin 13	*CLDN13*	GGG TGA CCT TTC CAG ATG ATG<br>CCA CCT GTT TTC CCG AAC CT
zonula occludens protein 1	*ZO-1*	TGA ACG CTC TCA TAA GCT TCG TAA<br>ACC GTA CCA ACC ATC ATT CAT TG
zonula occludens protein 2	*ZO-2*	ATG GGA GCA GTA CAC CGTG A<br>GCT GAAC GGC AAA CGA ATG G
zonula occludens protein 3	*ZO-3*	CTG TGG AGA ACG TCA CAT CTG<br>CGG GGA CGC TTC ACT GTA AC
connexin 43	*Cx43*	TTG GCT CAC GTG TTC TAT GT<br>ACC TCT CAT CTT CAC CTT GC
glyceraldehyde 3-phosphate dehydrogenase	*GAPDH*	CAT GGC CTC CAA GGA GTA AG<br>TGT GAG GGA GAT GCT CAG TG
ribosomal protein 13A	*RLP13A*	AGC CTA CCA GAA AGT TTG CTT AC<br>GCT TCT TCT TCC GAT AGT GCA TC

Table A3

gene name	gene ID	primer sequences (3`- 5`)
insulin growth factor 1	*IGF1*	GCT CTT CAG TTC GTG TGT GG<br>GCC TCC TTA GAT CAC AGC TCC
insulin growth factor 2	*IGF2*	GTG GCA TCG TTG AGG AGT G<br>CAC GTC CCT CTC GGA CTT G
B-actin	*BACT*	GAG AGG GAA ATC GTG CGT GA<br>ACA TCT GCT GGA AGG TGG AC
smooth muscle actin α	*aSMA*	AAA AGA CAG CTA CGT GGG TGA<br>GCC ATG TTC TAT CGG GTA CTT C
collagen 1A1	*COL1A1*	GTG CGA TGA CGT GAT CTG TGA<br>CGG TGG TTT CTT GGT CGG T
keratin 1	*KRT1*	AGA GTG GAC CAA CTG AAG AGT<br>ATT CTC TGC ATT TGT CCG CTT
connexin 43	*Cx43*	GTG ACT GGA GCG CCT TAG GCA A<br>AGG ACC CAG AAG CGC ACA TGA G
glyceraldehyde 3-phosphate dehydrogenase	*GAPDH*	AGG GCT GCT TTT AAC TCT GGT<br>CCC CAC TTG ATT TTG GAG GGA
ribosomal protein 13A	*RLP13A*	GCC CTA CGA CAA GAA AAA GCG<br>TAC TTC CAG CCA ACC TCG TGA

Table A4

oxPTM	m/z	target amino acids
didehydrogenation (-4H)	-4.03	A, C, I, K, L, M, P, Q, S, T, V, W, Y
dehydrogenation (-2H)	-2.01	A, D, F, G, H, I, L, P, R, S, T, V, Y, C, E, K, M, N, Q, W
amidation (+NH/-O)	-0.98	A, D, F, G, I, L, P, R, S, T, V, Y, C, E, K, M, N, Q, W
deamidation (+O/-NH)	0.98	A, D, F, G, H, I, L, P, R, S, T, V, C, E, K, M, N, Q, W
ring cleavage (+2O/-HCN)	4.97	H, W
carbonylation (+O/-2H)	13.97	A, D, F, G, H, I, L, P, S, T, V, Y, C, E, K, M, N, Q, W
oxidation (+O)	15.99	A, D, F, G, H, I, L, P, S, T, V, Y, C, E, K, M, N, Q, W
nitrosylation (+O/+N/-H)	28.99	A, C, I, K, M, N, P, Q, S, T, V, W, Y
quinonylation (+2O/-2H)	29.97	A, D, F, H, I, L, P, R, S, V, Y, C, E, K, M, Q, W
dioxidation (+2O)	31.98	A, D, F, G, H, I, L, P, S, T, V, Y, C, E, K, M, N, Q, W
chlorination (+Cl/-H)	33.96	A, D, F, G, H, I, L, R, S, V, Y, C, E, K, M, N, Q, W
nitration (+2O/+N/-H)	44.98	D, E, F, G, I, K, L, Q, S, V, W, Y
quinonylation+O (+3O/-2H)	45.96	C, D, E, F, I, K, M, N, P, Q, S, V, W, Y
trioxidation (+3O)	47.98	A, D, F, G, H, I, L, P, V, Y, C, E, K, M, Q, W
nitration+O (+3O/+N/-H)	60.97	A, D, F, G, I, L, P, S, V, Y, C, K, M, N, Q, W
nitration+2O (+4O/+N/-H)	76.97	A, D, F, G, I, L, P, S, V, Y, C, K, M, N, Q, W

Table A5

ID	protein name	% Ox	A	C	D	E	F	G	H	I	K	L	M	N	P	Q	R	S	T	V	W	Y
**P30101**	Protein disulfide-isomerase A3	5.9					1															
**P05997**	Collagen α2(V) chain	100													1							
**P02647**	Apolipoprotein A-I	6.3											6	1		1					4	
**P04259**	Keratin, type II cytoskeletal 6B	5.9										2	1	5								
**P16157**	Ankyrin-1	7.4										1		1								
**P07942**	Laminin subunit β1	100																		3		
**P01023**	α2-macroglobulin	7.4	1					2					4	17							1	
**P62258**	14-3-3 protein ε	10								1												
**P01011**	α1-antichymotrypsin	11.8				1							7	3								2
**P02042**	Hemoglobin subunit δ	39											39							1		1
**P07476**	Involucrin	4.8														1						
**P60174**	Triosephosphate isomerase	22.6						1						11								
**P0DP25**	Calmodulin-3	28.6												3					1			
**P55072**	Transit. endopl. reticulum ATPase	7.1					1															
**P60709**	Actin, cytoplasmic	16.1										1	12	12								2
**Q99497**	Parkinson's disease protein 7	11.1								1												
**P02730**	Band 3 anion transport protein	20.8						1					3	6								
**P02749**	β2-glycoprotein 1	12.5								1												
**P00918**	Carbonic anhydrase 2	22.1						1						14								
**P01859**	Immunoglobulin heavy constant γ	64.3												17							1	
**Q8N1N4**	Keratin, type II cytoskeletal 78	42.9										2							1			
**P01593**	Immunoglobulin κ variable 1D-33	16.7				1																
**P35908**	Keratin, type II cytoskeleton 2 epidermis	8.9											8	2		2	1					1
**P01602**	Immunoglobulin κ variable 1-5	50				1																
**P05164**	Myeloperoxidase	4.9											1	1		1						
**P31947**	14-3-3 protein sigma	4.5		1																		
**P30043**	Flavin reductase (NADPH)	17.4			1								5	2								
**P06733**	α-enolase	5.9								1				2								
**P06702**	Protein S100-A9	13							2			7	41	12		1						
**P13716**	δ-aminolevulinic acid dehydratase	5.9																		1		
**Q14624**	Inter-α-trypsin inhibitory heavy chain H4	7.5				1								2								
**P02763**	α1-acid glycoprotein 1	4.9											2								2	
**P01624**	Immunoglobulin κ variable 3-15	16.7												1				1				
**Q9NZT1**	Calmodulin-like protein 5	20						1						1					1			
**A0A0J9YY99**	Ig-like domain-containing protein	33.3										1		3								

76 *Figure A1*

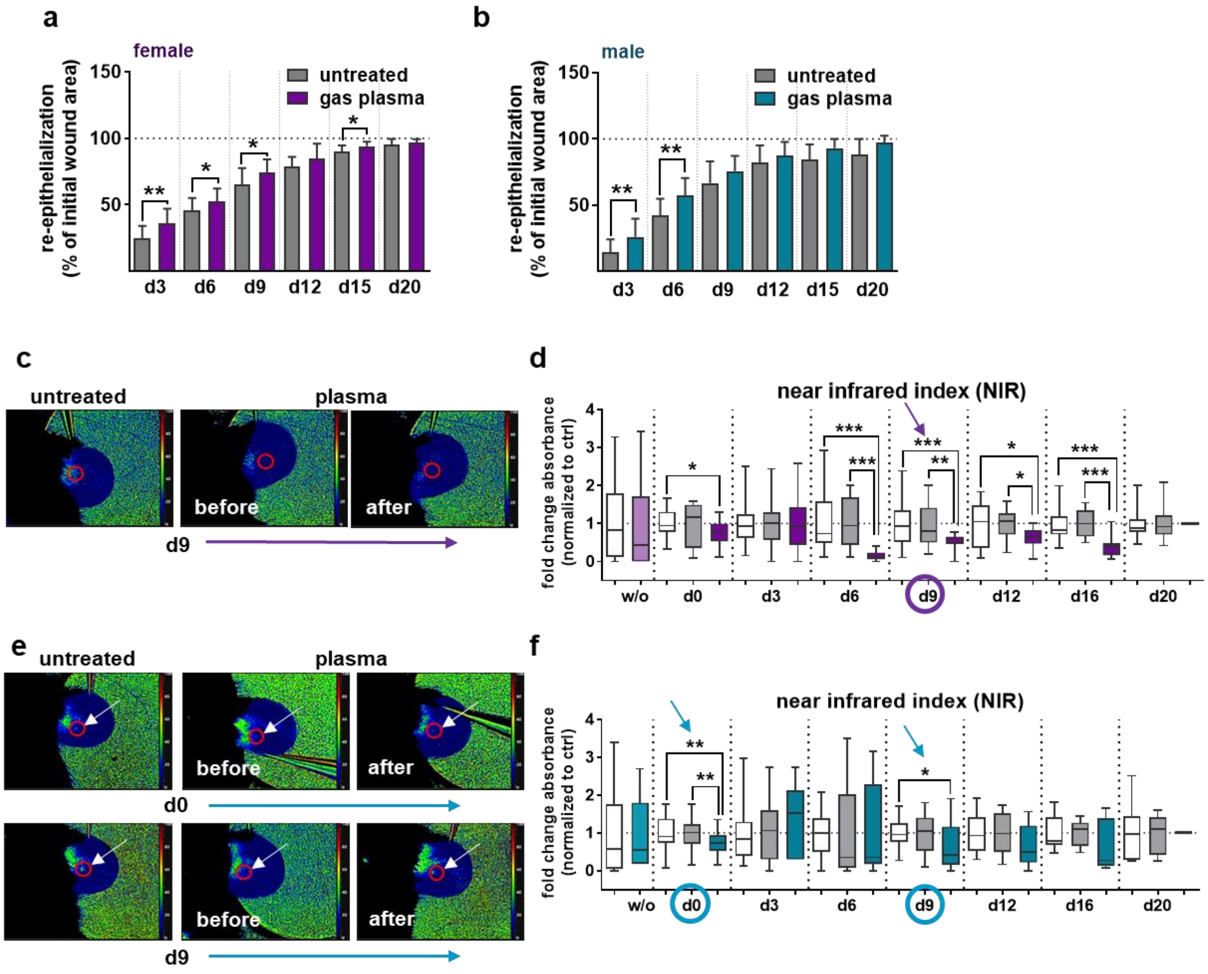

77

Figure A2

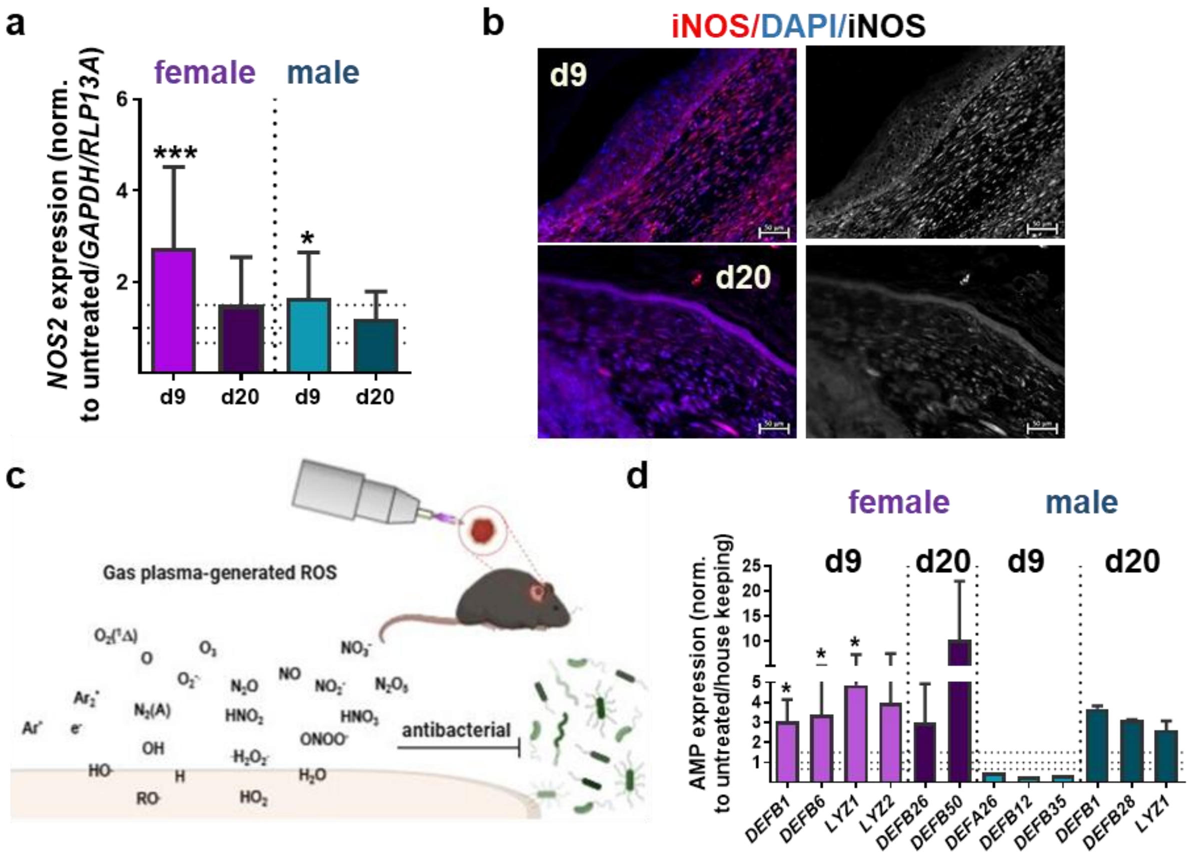
a
female
male
NOS2 expression (norm. to untreated/GAPDH/RLP13A)
6
4
2
***
*
d9
d20
d9
d20
b
iNOS/DAPI/iNOS
d9
d20
c
Gas plasma-generated ROS
antibacterial
d
female
male
d9
d20
d9
d20
AMP expression (norm. to untreated/house keeping)
25
20
15
10
5
4
3
2
1
DEFB1
DEFB6
LYZ1
LYZ2
DEFB26
DEFB50
DEFA26
DEFB12
DEFB35
DEFB1
DEFB28
LYZ1

Figure A3

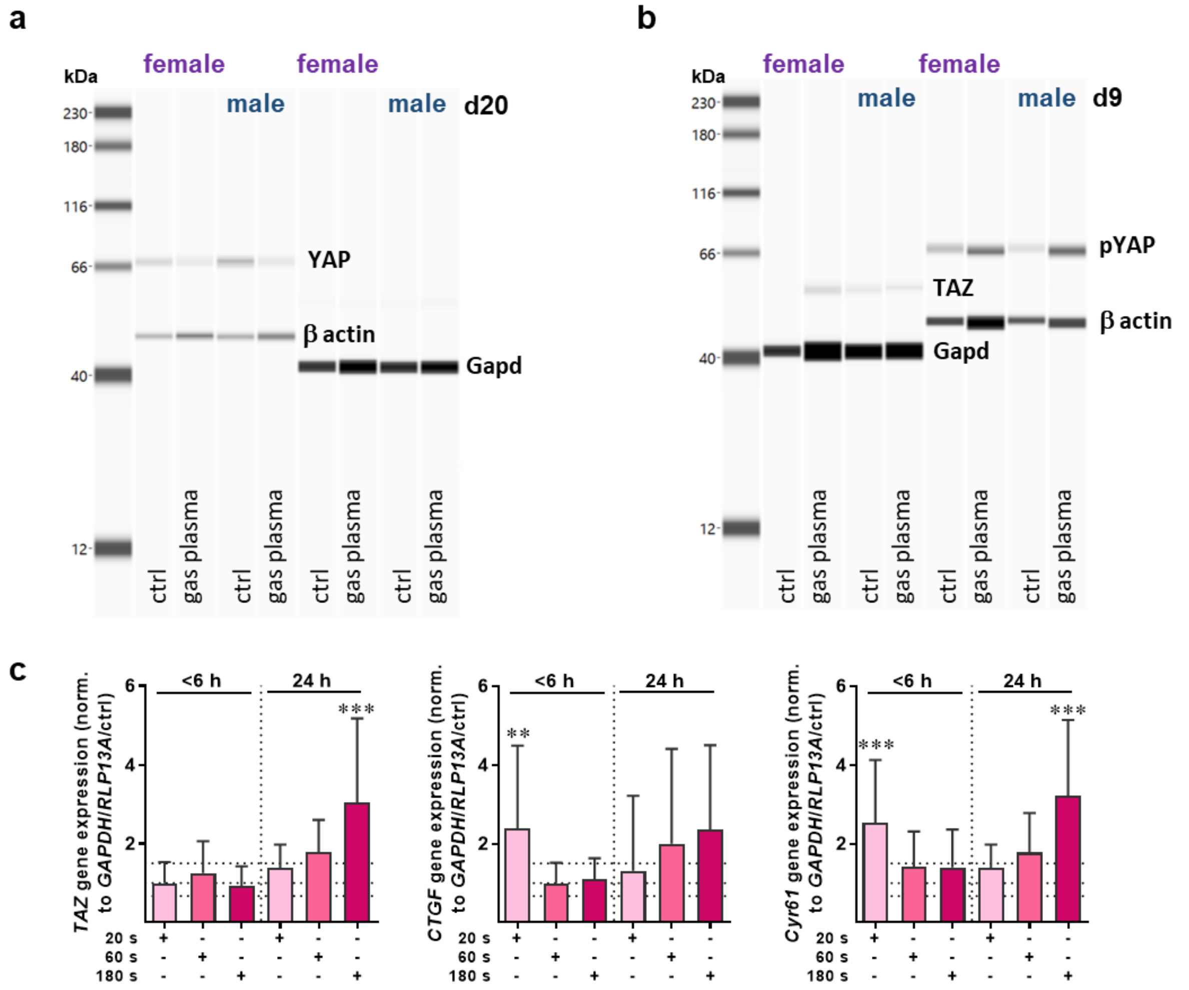
a
kDa
230
180
116
66
40
12
female
male
female
male
d20
YAP
β actin
Gapd
ctrl
gas plasma
b
pYAP
TAZ
d9
c
<6 h
24 h
TAZ gene expression (norm. to GAPDH/RLP13A/ctrl)
CTGF gene expression (norm. to GAPDH/RLP13A/ctrl)
Cyr61 gene expression (norm. to GAPDH/RLP13A/ctrl)
20 s
60 s
180 s
**
***

References

1 Wenske, S. *et al.* Reactive species driven oxidative modifications of peptides-Tracing physical plasma liquid chemistry. *J. Appl. Phys.* **129**, 193305 (2021). https://doi.org/10.1063/5.0046685

2 Wenske, S. *et al.* Nonenzymatic post-translational modifications in peptides by cold plasma-derived reactive oxygen and nitrogen species. *Biointerphases* **15**, 061008 (2020). https://doi.org/10.1116/6.0000529

3 Bruno, G. *et al.* On the Liquid Chemistry of the Reactive Nitrogen Species Peroxynitrite and Nitrogen Dioxide Generated by Physical Plasmas. *Biomolecules* **10**, 1687 (2020). https://doi.org/10.3390/biom10121687

4 Takai, E. *et al.* Chemical modification of amino acids by atmospheric-pressure cold plasma in aqueous solution. *Journal of Physics D-Applied Physics* **47**, 285403 (2014). https://doi.org/10.1088/0022-3727/47/28/285403
